# Comprehensive lipidome of human plasma using minimal sample manipulation by liquid chromatography coupled with mass spectrometry

**DOI:** 10.1002/rcm.9472

**Published:** 2023-02-09

**Authors:** Bebiana C. Sousa, Zulema Gonzalez Klein, Diane Taylor, Greg West, Aveline Neo Huipeng, Michael J. O. Wakelam, Andrea F. Lopez‐Clavijo

**Affiliations:** ^1^ Lipidomics Facility Babraham Institute, Babraham Research Campus Cambridge UK; ^2^ Centro de Biotecnología y Genómica de Plantas (CBGP), Instituto Nacional de Investigación y Tecnología Agraria y Alimentaria (INIA) Universidad Politécnica de Madrid (UPM) Madrid Spain; ^3^ Departamento de Biotecnología‐Biología Vegetal, Escuela Técnica Superior de Ingeniería Agronómica, Alimentaria y de Biosistemas Universidad Politécnica de Madrid (UPM) Madrid Spain

## Abstract

**Rationale:**

The present work shows comprehensive chromatographic methods and MS conditions that have been developed based on the chemical properties of each lipid subclass to detect low‐abundance molecular species. This study shows that the developed methods can detect low‐ and/or very‐low‐abundant lipids like phosphatidic acid (PA) in the glycerophospholipid (GP) method; dihydroceramide (dhCer) and dihydrosphingosine/sphinganine (dhSPB) in the sphingolipid (SP) method; and lysophosphatidic acid (LPA), LPI, LPG and sphingosine‐1‐phosphate (SPBP) in the lysolipid method.

**Methods:**

An optimised method for the extraction of lysolipids in plasma is used in addition to Folch extraction. Then, four chromatographic methods coupled with mass spectrometry using targeted and untargeted approaches are described here. Three of the methods use a tertiary pumping system to enable the inclusion of a gradient for analyte separation (pumps A and B) and an isocratic wash (pump C). This wash solution elutes interfering compounds that could cause background signal in the subsequent injections, reducing column lifetime.

**Results:**

Semi‐quantitative values for 37 lipid subclasses are reported for a plasma sample (NIST SRM 1950). Furthermore, the methods presented here enabled the identification of 338 different lipid molecular species for GPs (mono‐ and diacyl‐phospholipds), SPs, sterols and glycerolipids. The methods have been validated, and the reproducibility is presented here.

**Conclusions:**

The comprehensive analysis of the lipidome addressed here of glycerolipids, GPs, sterols and SPs is in good agreement with previously reported results, in the NIST SRM 1950 sample, by other laboratories. Ten lipid subclasses LPS, LPI, alkyl‐lysophosphatidic acid/alkenyl‐lysophosphatidic acid, alkyl‐lysophosphatidylethanolamine/alkenyl‐lysophosphatidylethanolamine, dhCer (d18:0), SPB (d18:1), dhSPB (d18:0) and SPBP (d18:2) have been detected using this comprehensive method and are uniquely reported here.

## 1 INTRODUCTION

The large diversity and dynamic range of lipids in biological systems pose a challenge to their analysis. The use of a single lipid extraction method and high‐throughput liquid chromatography–mass spectrometry (LC–MS) analysis of lipids has been widely applied.^1^, ^2^, ^3^, ^4^ However, most LC–MS lipidomics studies require detection of low‐abundant signalling lipids, which must have an adequate lipid extraction method, appropriate internal standard(s) (IS) selection, liquid/gas chromatography separation conditions, MS instrument type and polarity mode. Recent efforts have been made to address some of these issues with international recommendations for lipid analysis by the Lipid MAPS Consortium.^5^ Changes in the abundances of each lipid subclass should be considered, as well as abundance differences in molecular species within a lipid subclass, which can cause ion suppression during electrospray ionisation (ESI) before MS detection. LC is an important analytical chemistry technique that can reduce ion suppression during ESI by separating analytes in tandem. LC provides orthogonality to accurate mass assignments (<5 ppm) and/or to multiple reaction monitoring (MRM) experiments by correlating the elution time of the IS with the endogenous lipid subclasses, when hydrophilic interaction liquid chromatography (HILIC) is used.

HILIC separates lipid subclasses by their head group,^6^, ^7^, ^8^ whereas reversed phase (RP) enables separation based on changes in the acyl‐chain composition (acyl‐chain length and number of double bonds).^9^, ^10^, ^11^, ^12^, ^13^ A combination of HILIC and RP can be used to target particular lipid subclasses, but this can be expensive as multiple injections are required. Most high‐throughput methods rely on RP separation, reducing data acquisition time and cost, to quickly find associations between lipids and diseases.^10^, ^11^ Efforts have been made to develop comprehensive and high‐throughput methods for the analysis of large data sets using supercritical fluid chromatography, either with derivatisation by methylation^14^ or without it,^15^, ^16^ which has shown potential in pushing the lipidomics field forward. However, there are still some low‐abundant lipid subclasses, for example, phosphatidic acid (PA), lysophosphatidic acid (LPA), sphingosine‐1‐phosphate (SPBP) and lysophosphoserine (LPS), that are not separated or detected by high‐throughput approaches. PA and LPA analytes have been successfully separated using solid‐phase extraction (SPE) before MS, and SPBP has been detected using SPE with further derivatisation.^8^ The challenge to separate acidic lipids, such as LPA and PA, in HILIC methods is often their insolubility in most common hydrophobic mobile phase solutions causing precipitation in the column (blockage) and the subsequent requirement to replace the column.^15^

Separating acidic lipids using RP columns is equally challenging due to the high affinity of the lipids to the stationary phase, which has then been reported as peak tailing in column chromatography.^16^ In addition to the challenges with chromatographic separation, the absence of a robust extraction method for LPA and LPS is still an issue. Methanol extraction has been previously reported for LPA detection but includes the addition of HCl,^17^ which can cause degradation of LPC to LPA^18^ and LPS to LPA.^19^ Citric acid and disodium hydrogen phosphate (pH 4.0) within butanol extraction have also been used for LPA and SPBP quantification.^18^ Derivatisation before LC has also been applied to low‐abundant lipids, such as SPBP^8^ to increase sensitivity (25‐ to 30‐fold). Here, samples for lysolipids (including LPA and LPS) and SPBP analyses were subjected to liquid–liquid extraction without derivatisation or enrichment, using an adaptation of the butanol method.^18^ The lipid extract contains a complex mixture of different lipid subclasses; nonetheless, robust repeatable chromatographic separation using an RP column was obtained for lysolipids, SPBP and dihydrosphingosine‐1‐phosphate (dhSPBP). The system uses mobile phases A and B to separate the analytes of interest, whereas solutions C and D remove other abundant lipids and compounds, thus increasing column lifetime with little to no carryover.

The LC system was also used with a HILIC column to increase the number of glycerophospholipid (GP) subclasses and sphingomyelin (SM) analysed in this comprehensive lipidomics study. GP includes PA, phosphatidylinositol (PI), phosphatidylcholine (PC), phosphatidylethanolamine (PE), phosphatidylglycerol (PG), phosphatidylserine (PS) and cardiolipin (CL) separated after Folch extraction (see Table 1). The Folch lipid organic extract also contains triacylglycerol (TG), diacylglycerol (DG), cholesterol (ST) and cholesteryl esther (CE) among other lipid subclasses. TG, DG, ST and CE are separated by an RP column and cannot be analysed in negative mode in contrast to GP. This family of lipids, also called neutral lipids, is characterised as charge‐neutral compounds. Neutral lipids need to be ionised for detection by a mass spectrometer, with cationisation being the most commonly used ionisation process for its identification.^20^ Cationisation with lithium, sodium, potassium acetate, sodium carbonate and sodium phosphate are some of the compounds capable of creating such ion adducts.^20^ However, the use of cations during chromatographic separation affects both chromatographic resolution and ionisation efficiency, in particular if phosphate is used. Ammonium formate or ammonium acetate has been used as an additive to aid in neutral lipid ionisation, with the advantage that TG and CE ammonium adducts have facile decomposition reactions under CID conditions.^20^

**TABLE 1:** Lipid classes, subclasses and shorthand notation used for each lipid class measured here

Diacylglycerophospholipids (GP)	Glycerolipids(GL), cholesterol and steryl esters (neutral lipids)	Monoacylglycerophospholipids (lysolipids)	Sphingolipids(SP)
Phosphatidic acid (PA)	Diacylglycerols (DG)	Lysophosphatidic acid (LPA)	Ceramide (Cer)
Phosphatidylcholine (PC)	Triacylglycerols (TG)	Lysophosphocholine (LPC)	Dihydroceramide (dhCer)
Phosphatidylethanolamine (PE)	Cholesterol (ST)	lysophosphoethanolamine (LPE)	Sphingosine (SPB)
Phosphatidylinositol (PI)	Cholesteryl ester (CE)	Lysophosphoglycerol (LPG)	dihydrosphingosine/sphinganine (dhSPB)
Phosphatidylserine (PS)	Alkyl‐diacylglycerol (O‐DG)	Lysophosphoinositol (LPI)	Sphingomyelin (SM)
Phosphatidylglycerol (PG)	Alkyl‐triacylglycerol(O‐TG)	Lysophosphoserine (LPS)	Dihydrosphingomyelin (dhSM)
Cardiolipin (CL)		Alkyl‐lysophosphatidic acid (O‐LPA)	Sphingosine‐1‐phosphate (SPBP)
alkyl‐acylglycerophosphocholine (O‐PC)		Alkenyl‐lysophosphatidic acid (P‐LPA)	Dihydrosphingosine‐1‐phosphate (dhSPBP)
Alkyl‐acylphosphatidylethanolamine (O‐PE)		Alkyl‐lysophosphatidylcholine (O‐LPC)	
Alkenyl‐acylglycerophosphoethanolamine (P‐PE)		Alkenyl‐lysophosphatidylcholine(P‐LPC)	
alkenyl‐acylglycerophosphocholine (P‐PC)		Alkyl‐lysophosphatidylethanolamine(O‐LPE)	
		Alkenyl‐lysophosphatidylethanolamine (P‐LPE)	

The use of ammonium in the mobile phase aids in chromatographic separation, but the spectrum also shows the presence of sodium adducts (see Figures S1 and S2 [supporting information]). Therefore, the method presented here relies on the quaternary pumping system for post‐column addition of aqueous ethylamine to displace sodium and ammonium adducts. Sodium and ammonium displacement is recommended due to the possible presence of lipid species with the same *m/z*, which could lead to over‐quantifying the relative amount of lipids present in a sample (see Table S1 and Figure S3 [supporting information]). This method also allows detection of ST by switching off the post‐column addition between 8 and 10 min. Other cholesterol metabolites (oxysterols) are not distinguishable using the method presented here. ST and oxysterol are analysed using GC–MS or LC–MS/MS, which require extensive sample preparation.^21^, ^22^, ^23^ Chromatographic separation of GP and neutral lipids using positive/negative ion switching in a single chromatographic method^24^ has been reported, and although this is a time‐saving approach, low‐abundant GPs like LPI, LPG and LPS are not observed.^9^, ^25^

Comprehensive lipid profiling also involves analysis of sphingolipid (SP) subclasses. Therefore, in addition to extractions using butanol for SPBP and Folch for SM, isopropanol:ethyl acetate solution was used for sphingoid‐based lipids like ceramides (Cer), dihydroceramides (dhCer), sphingosine (SPB) and dihydrosphingosine/sphinganine (dhSPB). Separation of Cer, dhCer, SPB and dhSPB lipid subclasses was achieved using an RP column and changing the proportions of mobile phases A and B. Once 100% B was reached, a switch valve event was introduced to enable elution of abundant GP, glycerolipids (GL) and SM by a gradient using pumps C and D. Most SP analysis relies on the mild hydrolysis of GPs before LC separation,^26^ so here we have reduced sample manipulation while still maintaining comprehensive lipidome analysis. Thus, four LC‐validated methods for the separation and detection of 37 lipid subclasses and their molecular species using a combination of HILIC and RP with a quaternary pumping system are presented here (Table 1).

**FIGURE 1:**
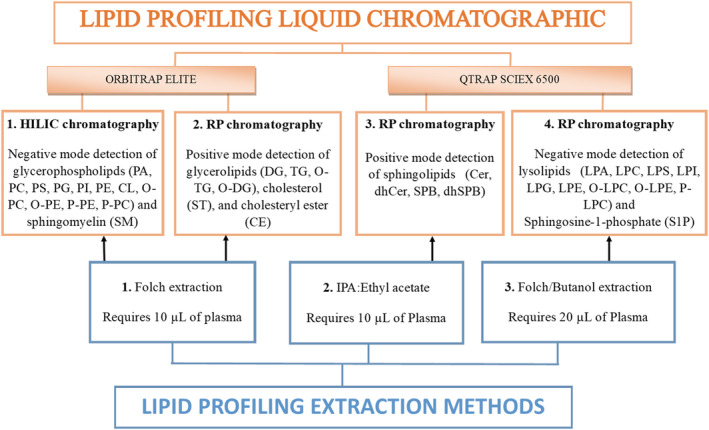
Experimental workflow for comprehensive lipid profile analysis in plasma includes phosphatidic acid (PA), phosphatidylcholine (PC), phosphatidylinositol (PI), phosphatidylserine (PS), phosphatidylethanolamine (PE), alkylacylglycerophosphoethanolamine (O‐PE), alkenyl‐acylglycerophosphoethanolamine (P‐PE), alkyl‐acylglycerophosphocholine (O‐PC), alkenyl‐acylglycerophosphocholine (P‐PC), cardiolipin (CL), lysophosphocholine (LPC), lysophosphoethanolamine (LPE), lysophosphatidic acid (LPA), lysophosphoglycerol (LPG), lysophosphoinositol (LPI), lysophosphoserine (LPS), cholesterol (ST), diacylglycerols (DG), triacylglycerols (TG), alkyl‐diacylglycerol (O‐TG), alkyl‐diacylglycerol (O‐DG), cholesteryl ester (CE), sphingosine (SPB), dihydrosphingosine/sphinganine (dhSPB), sphingosine‐1‐phosphate (SPBP), dihydrosphigosine‐1‐phosphate (dhSPBP), ceramides (Cer), dihydroceramides (dhCer), sphingomyelin (SM) and dihydrosphingomyelin (dhSM) [Color figure can be viewed at wileyonlinelibrary.com]

Figure 1 shows the experimental workflow used here for comprehensive lipid profile analysis using a Folch‐butanol extraction method suitable for lysolipids, SPBP and dhSPBP. Here, semi‐quantitative values for 37 lipid subclasses are reported for an SRM plasma sample (NIST SRM 1950).^27^, ^28^ The level of detail provided by this comprehensive analysis enabled the identification of 338 different lipid molecular species across the 44 lipid subclasses identified in human plasma. Reproducibility was tested using four replicates (run on different days).

An Orbitrap Elite mass spectrometer (Thermo Fisher Scientific, Waltham, MA, USA) and a TripleQuad 6500 (Sciex, Warrington, UK) mass spectrometer were used hyphenated to LC systems. The sensitivity provided by the TripleQuad with the added dissociation to specific acyl‐chain fragments coupled to the orthogonal selectivity of the LC detection enabled quantitation of low‐abundant signalling lipids (LPA, LPI, LPS, LPG, SPB and SPBP). Abundant lipid subclasses were detected and quantified using the Orbitrap mass spectrometer, with mass accuracy assignments ≤5 ppm and the retention time orthogonality provided by chromatographic separation.

## 2 EXPERIMENTAL

### 2.1 Chemicals and samples

Plasma samples from the National Institute of Standards and Technology (NIST, Gaithersburg, MD, USA) were used. The material SRM 1950 represented ‘normal’ human plasma NIST widely used for QC (full description of this material is provided in its certificate of analysis, www.nist.gov/srm). Ultra‐purity (UpS)‐grade acetonitrile, chloroform, methanol, isopropanol, hexane, dichloromethane, ethyl acetate and formic acid were obtained from ROMIL Ltd (Waterbeach, Cambridge, UK). Super‐purity‐grade (SpR) ammonium acetate was obtained from ROMIL Ltd. Butanol (HPLC grade), ammonium formate and ethylamine were purchased from Sigma‐Aldrich (Dorset, UK). Milli‐Q water was obtained using a Milli‐Q Milli pore system (Milli‐Q plus 185).

### 2.2 Internal standards

The ISs were acquired from Avanti, and their concentrations are presented in Table S2 (supporting information). LIPID MAPS nomenclature and shorthand notation^29^ were used throughout this manuscript. ISs were diluted with chloroform:methanol (1:1) (v/v) before spiking into the aqueous‐methanolic phase before extraction to relatively quantify each molecular species. Recovery defined as area ratio of the extracted analyte in a matrix (plasma) compared to the unextracted analyte in plasma against the area of the ISs is not used in relative quantification of lipid endogenous species. The reasons are as follows. First, the analyte corresponds to each endogenous molecular species observed, and it will be prohibitively expensive to have an IS for each molecular species. Second, the different natural occurring abundances of each molecular species within each lipid subclass (see profiles shown in Figures S6–S11 [supporting information]) will require different IS at different concentrations. Parallelism could be used for endogenous lipid analysis using a surrogate matrix (e.g., serially diluted plasma or phosphate‐buffered saline) and applying the definition of recovery. However, parallelism is not employed here.

### 2.3 Lipid extraction using Folch method and Folch‐butanol method

Ten microlitres of human plasma was subjected to Folch extraction method^20^ for analysis of GPs, GLs and SPs (Cer, dhCer, SPB, dhSPB and SM); 20 μl of human plasma was used for the extraction of lysolipids and SPBP using the Folch‐butanol method (see Section S1 [supporting information]). Each sample was extracted with the mixture of ISs per each lipid subclass to relatively quantify lipid levels. After extraction and before analysis, the lipid mixtures were resuspended in either 50 μl of chloroform:methanol (1:1) for the lipid species extracted using the Folch method or acidified methanol for those extracted using the Folch‐butanol method.

### 2.4 Lipid extraction using IPA:ethyl acetate method

A mixture of IPA:ethyl acetate (570 μl, 8:2 v/v) was added to 10 μl of plasma spiked with 10 μl of IS. The samples were mixed for 10 min and centrifuged for 10 min at 3000*g* using the procedure developed for absolute quantitation of four Cer molecular species (Cer d18:1/16:0, Cer d18:1/18:0, Cer d18:1/24:0 and Cer d18:1/24:1).^29^

### 2.5 LC–MS analysis

Four methods were developed for the separation and detection of 32 lipid subclasses and their molecular species. The lipids studied belong to the lipid classes of GPs, GLs, sterols (ST) and SPs, which are presented in Table 1. GL, ST and CE are also known as neutral lipids, whereas plasmanyl (O‐lipids) and plasmenyl (P‐lipids) are called ether lipids. A summary of each method, with the corresponding lipid subclasses measured, extraction method, chromatographic column, mass spectrometer and ionisation mode, is presented in Table 2.

**TABLE 2:** LC–MS or LC–MS/MS methods

Method name	Lipid subclasses measured	Extraction method	Chromatographic column dimensions	Mass spectrometer type	Ionisation polarity
GP and SM	PA, PC, PE, PI, PS, PG, CL, O‐PC, P‐PC, O‐PE, P‐PE, SM, dhSM	Folch	Cogent silica C (2.1 × 150 mm, 4 μm)	Orbitrap Elite	Negative
GL and ST	DG, TG, ST, CE, O‐DG, O‐TG	Folch	XSelect CSH C18 (2.1 × 150 mm, 2.5 μm)	Orbitrap Elite	Positive
Lysolipids and SPBP	LPA, LPC, LPE, LPS, LPI, LPG, O‐LPC, P‐LPC, O‐LPE, P‐LPE, O‐LPA, P‐LPA, SPBP, dhSPBP	Folch‐butanol	Phenomenex EVO C18 (2.1 × 100 mm, 2.6 μm)	QTrap 6500	Negative
Ceramides and SPB	Cer, dhCer, SPB, dhSPB	IPA:ethyl acetate	Agilent Zorbax 300 SB (2.1 × 50 mm, 1.8 μm)	QTrap 6500	Positive

Abbreviations: Cer, ceramide; CL, cardiolipin; DG, diacylglycerol; dhSPBP, dihydrosphingosine‐1‐phosphate; dhSM, dihydrosphingomyelin; GL, glycerolipid; GP, glycerophospholipid, LC–MS, liquid chromatography–mass spectrometry; LPA, lysophosphatidic acid; LPI, lysophosphoinositol; LPS, lysophosphoserine; O‐DG, alkyl‐diacylglycerol; O‐LPA, alkyl‐lysophosphatidic acid; O‐LPC, alkyl‐lysophosphatidylcholine; O‐LPE, alkyl‐lysophosphatidylethanolamine; O‐PC, alkyl‐acylglycerophosphocholine; O‐PE, alkyl‐acylphosphatidylethanolamine; O‐TG, alkyl‐triacylglycerol; PA, phosphatidic acid; PC, phosphatidylcholine; PE, phosphatidylethanolamine; PG, phosphatidylglycerol; PI, phosphatidylinositol; PS, phosphatidylserine; P‐LPA, alkenyl‐lysophosphatidic acid; P‐LPC, alkenyl‐lysophosphatidylcholine; P‐LPE, alkenyl‐lysophosphatidylethanolamine; P‐PC, alkenyl‐acylglycerophosphocholine; P‐PE, alkenyl‐acylglycerophosphoethanolamine; PS, phosphatidylserine; SM, sphingomyelin; SPBP, sphingosine‐1‐phosphate; TG, triacylglycerol.

#### 2.5.1 GPs and SM

Phospholipid molecular species from PA, PC, PE, PI, PS, PG, CL, alkyl‐acylglycerophosphocholine (O‐PC), alkenyl‐acylphosphatidylcholine (P‐PC), alkyl‐acylphosphatidylethanolamine (O‐PE), alkenyl‐acylglycerophosphoethanolamine (P‐PE), dihydrosphingomyelin (dhSM) and SM molecular species were detected in negative ionisation mode. These lipid subclasses were separated by high‐performance liquid chromatography (HPLC) using a Cogent Silica C column (2.1 × 150 mm, 4 μm) on a Nexera XR 20 AD system (Shimadzu, Kyoto, Japan) coupled to an Orbitrap Elite mass spectrometer (Thermo Fisher Scientific). Mobile phase A was isopropanol/hexane/2 M ammonium acetate 58:40:2 (v/v), whereas mobile phase B was isopropanol/hexane/10mM ammonium acetate 50:40:10 (v/v) supplemented with 0.1% formic acid. Mobile phase C, isopropanol/dichloromethane 90:10 (v/v), was used to elute other lipid subclasses after 25.0 min of gradients A and B. See Section S2.1 (supporting information) for detailed LC–MS conditions.

#### 2.5.2 GLs and sterols

DGs, TGs, cholesterol (ST), cholesteryl ester (CE), alkyl‐diacylglycerol (O‐DG) and alkyl‐triacylglycerol (O‐TG) were detected in positive mode with post‐column addition of aqueous ethylamine solution (0.08%) during the elution gradient to favour ionisation and MS detection of TG, DG, O‐DG, O‐TG and CE. As these lipids are charge‐neutral compounds, adduct formation is needed for ionisation.^20^ Therefore, ethylamine adducts [M + C_2_H_7_]^+^ are formed after chromatographic separation but before MS analysis to avoid affecting the solubility of the compound and the separation in the column.^20^ Chromatographic separation was achieved using an Xselect CSH C18 column (2.1 × 150 mm, 2.5 μm) in a Nexera XR 20 AD system (Shimadzu) coupled to an Orbitrap Elite mass spectrometer (Thermo Fisher Scientific). Mobile phase A consisted of acetonitrile and water (60:40, v/v), and mobile phase B consisted of isopropanol and acetonitrile (90:10, v/v); both 10mM ammonium formate and 0.1% formic acid were used as additives. Mobile phase C line was replumbed for C to work as a post‐column addition of 0.08% ethylamine in water. Mobile phase D, composed of 40% isopropanol and acetonitrile (90:10, v/v) with 10mM ammonium acetate and 60% isopropanol/hexane/ethyl acetate/acetonitrile/dichloromethane (4:3:1:1:1 v/v), was used to wash the column after sample injection. Detailed LC–MS conditions are presented in Section S2.2 (supporting information).

#### 2.5.3 Lysolipids and SPBP

LPA, lysophosphocholine (LPC), lysophosphatidylethanolamine (LPE), lysophosphoglycerol (LPG), LPI, LPS, alkyl‐lysophosphatidic acid (O‐LPA), alkenyl‐lysophosphatidic acid (P‐LPA), alkyl‐lysophosphatidylcholine (O‐LPC), alkenyl‐lysophosphatidylcholine (P‐LPC), alkyl‐lysophosphatidylethanolamine (O‐LPE), and SPBP molecular species were ionised in negative mode, and chromatographic separation was achieved using a Prominence 20 AD system (Shimadzu) with a Phenomenex EVO C18 column (2.1 × 100 mm, 2.6 μm) coupled to a QTRAP 6500 mass spectrometer (Sciex). Mobile phase A consisted of an aqueous solution of 5mM ammonium formate, whereas B consisted of 5mM ammonium formate in acetonitrile; 0.5% formic acid was added to mobile phases A and B. Section S2.3 (supporting information) presents detailed LC–MS conditions.

#### 2.5.4 Cer and SPB

Cer, dihydroceramide (dhCer), SPB and dihydrosphingosine/sphinganine (dhSPB) molecular species were separated in an RP column (Agilent Zorbax 300 SB, 2.1 × 50 mm, 1.8 μm) and detected using a QTRAP 6500 mass spectrometer. Mobile phase A consisted of 5mM ammonium acetate in water, and mobile phase B consisted of acetonitrile with 0.4% formic acid. Mobile phase C consisted of 60% acetonitrile with 0.1% formic acid and 40% 10mM ammonium acetate in water. Mobile phase D consisted of 60% isopropanol/hexane/ethyl acetate/acetonitrile/dichloromethane (4:3:1:1:1 v/v) and 40% isopropanol/acetonitrile (90:10, v/v) with 10mM ammonium acetate. The gradient and mass spectrometer conditions are presented in Section S2.4 (supporting information).

### 2.6 Data processing and relative quantification

Data acquired by the Orbitrap mass spectrometer were processed using a Lipid Data Analyser (Graz University of Technology, version 2.6.3).^30^ Relative quantification of each individual molecular lipid species was given as the ratio of the area of the extracted ion count (EIC), of a given *m/z* value, to the area of EIC of the IS, for each lipid subclass. The EIC was also aligned to the retention time (RT) of the standard for orthogonal detection in the case of HILIC chromatography. RP methods were based on the RT of the IS where greater RTs were expected for longer acyl‐chain moieties, and the presence of one or more double bonds for a particular acyl‐chain length would result in earlier elution when compared to the saturated species. Data acquired by QTRAP 6500 were manually processed using Sciex Multiquant (MQ) software (version 3.0.3). MQ calculated the area ratio of each molecular species against the IS. The results were then analysed using an in‐house R‐code^31^ (RStudio, PBC, Boston, MA, USA) (http://www.rstudio.com/) to obtain the quantities (ng) of each lipid normalised to the volume of the sample (μl).

### 2.7 Validation

Linearity, limit of detection (LOD) and limit of quantitation (LOQ) of the four methods presented in Section 2.4 were determined based on the analysis of SRM 1950 plasma spiked with each IS at at least six concentrations. The concentration points for the calibration curve were defined between 0 and 12 ng/μl using the ISs of GP, SM, GL and CE. LPA, LPS, LPE, LPG and LPI calibration curve points ranged from 0 to 0.5 ng/μl, SPBP from 0 to 1.0 ng/μl, LPC from 0 to 0.4 ng/μl and cholesterol from 0 to 60 ng/μl. Linearity was determined using calibration curves plotting peak areas against IS concentration (see Figure S3 [supporting information]), and LOD and LOQ were determined from signal‐to‐noise ratios, which were 3 and 10, respectively.^32^ Reproducibility, as the standard deviation, was determined by consecutive injections of four replicates of SRM 1950 plasma extracted on different days. Matrix effects were calculated as the ratio of the abundance of the pure IS to the IS spiked into SRM 1950 plasma.

## 3 RESULTS AND DISCUSSION

### 3.1 Validation of the chromatographic methods in SRM 1950 plasma

**FIGURE 2:**
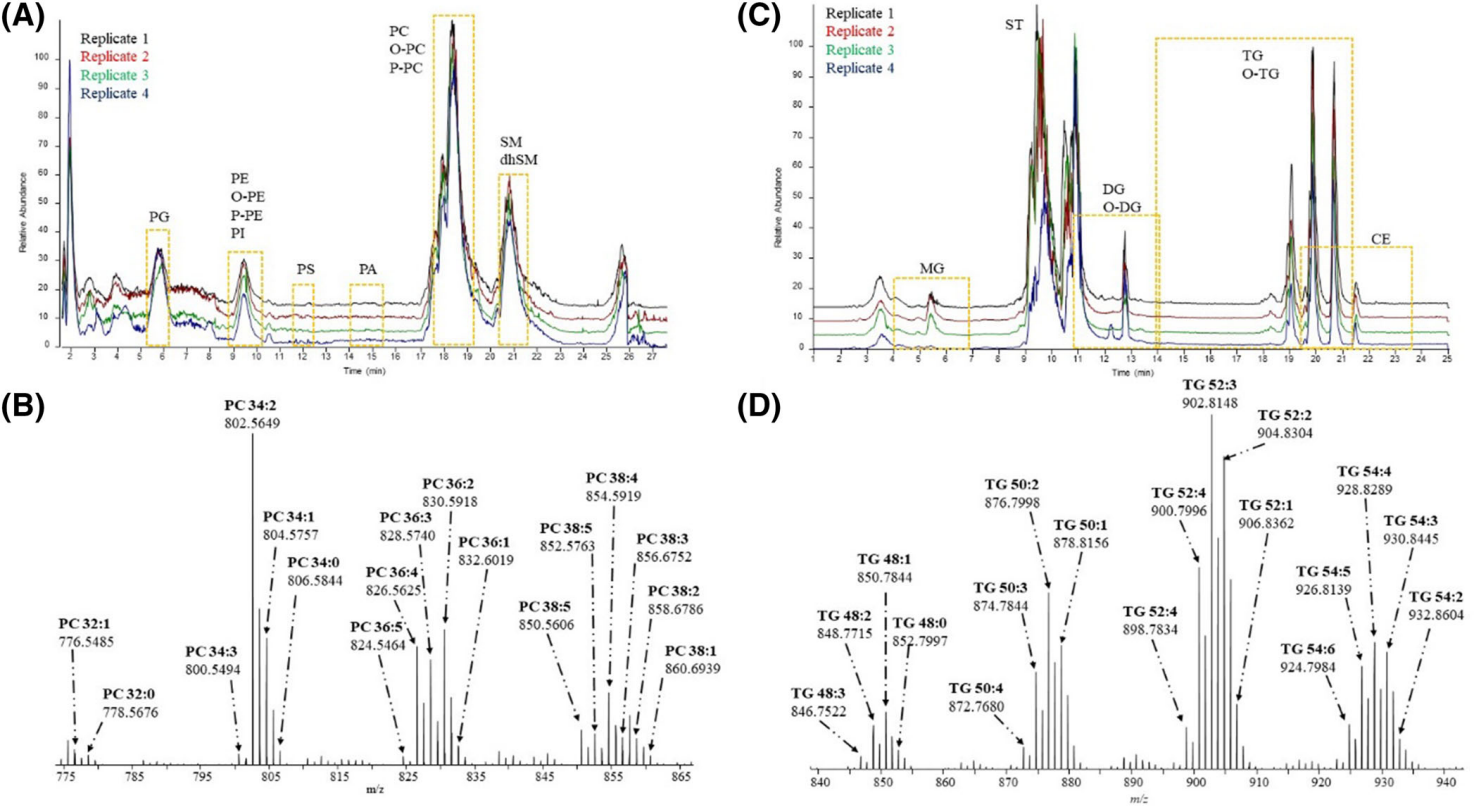
LC–MS (liquid chromatography–mass spectrometry) analysis of SRM 1950 samples. A, Total ion count showing the LC separation of phosphoglycerolipids (phosphatidylinositol [PI], phosphatidylcholine [PC], phosphatidylethanolamine [PE], phosphatidylglycerol [PG], phosphatidylserine [PS] and sphingolipids (sphingomyelin [SM] and dihydrosphingomyelin [dhSM]) using a HILIC (hydrophilic interaction liquid chromatography) column. B*,* Spectrum of PC lipid subclass molecular species co‐eluting at the retention time of the internal standard, 17.99 min. C, Total ion count showing the LC separation of the neutral lipids (diacylglycerols [DG], triacylglycerols [TG], cholesterol [ST] and cholesteryl esters [CE] using the RP (reversed‐phase) method. The yellow box highlights the time for the elution of the subclass species starting from the saturated short acyl‐chain species to the last long acyl‐chain moiety, including the molecular species with double bonds (unsaturations) in between. D, Sum of the spectrum obtained for the TG lipid subclass molecular species, which eluted between 17.5 and 21 min, including the retention time of the internal standard TG 51:1 at 19.41 min [Color figure can be viewed at wileyonlinelibrary.com]

Figure 2A shows the total ion count (TIC) from the chromatographic separation of the different glycerophosholipid subclasses (PG, PS, PA, PE, PC, PI, O‐PE, P‐PE, P‐PC and O‐PE) and SM. PG molecular species eluted first at 5.5 min, followed by PE (8.73 min) and PI (9.43 min). PS and PA separated at 12.0 and 15.0 min, respectively. Liquid chromatographic separation of low‐abundance PA and abundant PS molecular species has previously been reported as challenging (10.1016/j.ab.2007.12.027). It was important in the development of this LC method that PA eluted at a different time to PS and PC because both PS and PC can decompose in the source to form PA. As PA accounts for less than 0.1% of the total glycerophospholipids (GP) concentration in human plasma,^33^ endogenous PA molecular species were not at a detectable level in the 10 μl plasma analysed. Moreover, PA and PS were not reported in SRM 1950 using high‐throughput methods, so analysis using the more sensitive QTrap instrument was not performed.^34^ Nonetheless, the added ISs of both PA 35:1 and PS 35:1 were detected in the SRM 1950 replicates at the RT, shown in Figure S4A (supporting information). Similarly, CL was not previously reported in SRM 1950 as a consensus value,^34^ with CL 56:0 IS eluting using the chromatographic conditions employed here.

Figure 2A also shows the most abundant peak at 18.0 min that corresponds to the elution of PC. The spectrum of PC molecular species (Figure 2B) shows acyl chains with 32, 34, 36 and 38 carbon atoms in the SRM 1950 sample. These numbers of carbon atoms correspond to the sum composition^35^ of the acyl chains located in both positions (*sn*‐1 and *sn*‐2) on the glycerol moiety. Figure 2B shows that the most abundant PC molecular species in the spectrum is PC 34:2, which is likely to represent the acyl composition of 16:0/18:2; 34:2 acyl‐chain composition is considered the most likely acyl composition reported in mammalian tissue in brain and white matter of mammals, with the saturated chain in *sn‐1* position and the longer, unsaturated chain in *sn‐2* position.^36^, ^37^ Results reported for SRM 1950 using Folch extraction and RP nano‐column observed the presence of PC 34:2,^38^ but PC 34:2 was not the most abundant molecular species within the phosphatydylcholine subclass as a consensus species reported by Bowden et al.^39^ Other studies using MeOH and methyl‐*tert*‐butyl ether with a high‐throughput RP column neither detected nor reported PC 34:2.^38^ The absence of PC 34:2 might be attributed to the lipid extraction method employed instead of the use of a high‐throughput RP column. Using the method presented here, PC 34:3 and PC 34:4 molecular species were also observed in the spectrum (Figure 2B). These particular molecular species were not reported using Folch extraction and an RP nano‐column by Lee et al.^38^

PC and PE eluted (shown in Figure 2A) at similar RTs to plasmenyl (P‐lipids) and plasmanyl (O‐lipids) with choline (P‐PC, O‐PC) and ethanolamine (P‐PE, O‐PE) head groups, because HILIC separation is achieved based on the head group and not on the acyl‐chain composition. Separation of plasmenyl and plasmanyl lipid subclasses still remains a challenge, and it is reported here as the P‐/O‐ choline and ethanolamine species (Table S5 [supporting information]). Other lipid subclasses separated using the HILIC method presented here are SM and PI. Interestingly, SM and PI of consensus (harmonised) lipid subclasses reported by Bowden et al^39^ show a similar abundance distribution to the one observed using the comprehensive approach here (see Figures S4 and S5 [supporting information]). In addition, five PG molecular species were identified here in SRM 1950; however, only PG 34:1 was reported as a consensus by all the participant laboratories by Bowden and co‐authors.^39^ A similar trend is observed for DG, where five DG molecular species were common to at least five laboratories, whereas the RP method reported here assigned four additional DG species (see Table S5 [supporting information]).

Figure 2C also shows cholesterol (ST) eluting at 9.60 min, a time when post‐column addition is switched off, which aligns with the RT of the deuterated internal standard (d6‐ST) at 9.51 min. DG, TG, O‐DG, O‐TG and CE molecular species were detected as ethylamine adducts [M + C_2_H_7_N]^+^. DG is the least abundant neutral lipid subclass in mammals^40^; therefore, separation from high‐abundant TG, CE and ST is key for its analysis. The yellow areas highlighted in Figure 2C correspond to the elution of the whole *m/z* range of molecular species for each subclass as RP separates the lipids by the acyl‐chain composition. Interestingly, the yellow areas in Figure 2C show little overlap between the lipid subclasses DG/O‐DG and TG/O‐TG. Figure 2C also shows that some CE and TG species co‐elute, whereas abundant GPs elute at 11 min. The spectrum for TG containing 50, 52 and 54 carbon atoms is shown in Figure 2D. TG molecular species with 56 and 58 carbon atoms were also observed using the comprehensive method reported here, with an additional 24 molecular species not observed by Bowden et al.^39^ TG 52:3 is the most abundant molecular species among the TG commonly found in the SRM 1950 plasma sample^39^ (Figure S7 [supporting information]). Moreover, CE profile of abundances and molecular species reported here coincides with the consensus species observed by Bowden and co‐authors^39^ (see Figure S8 [supporting information]).

**FIGURE 3:**
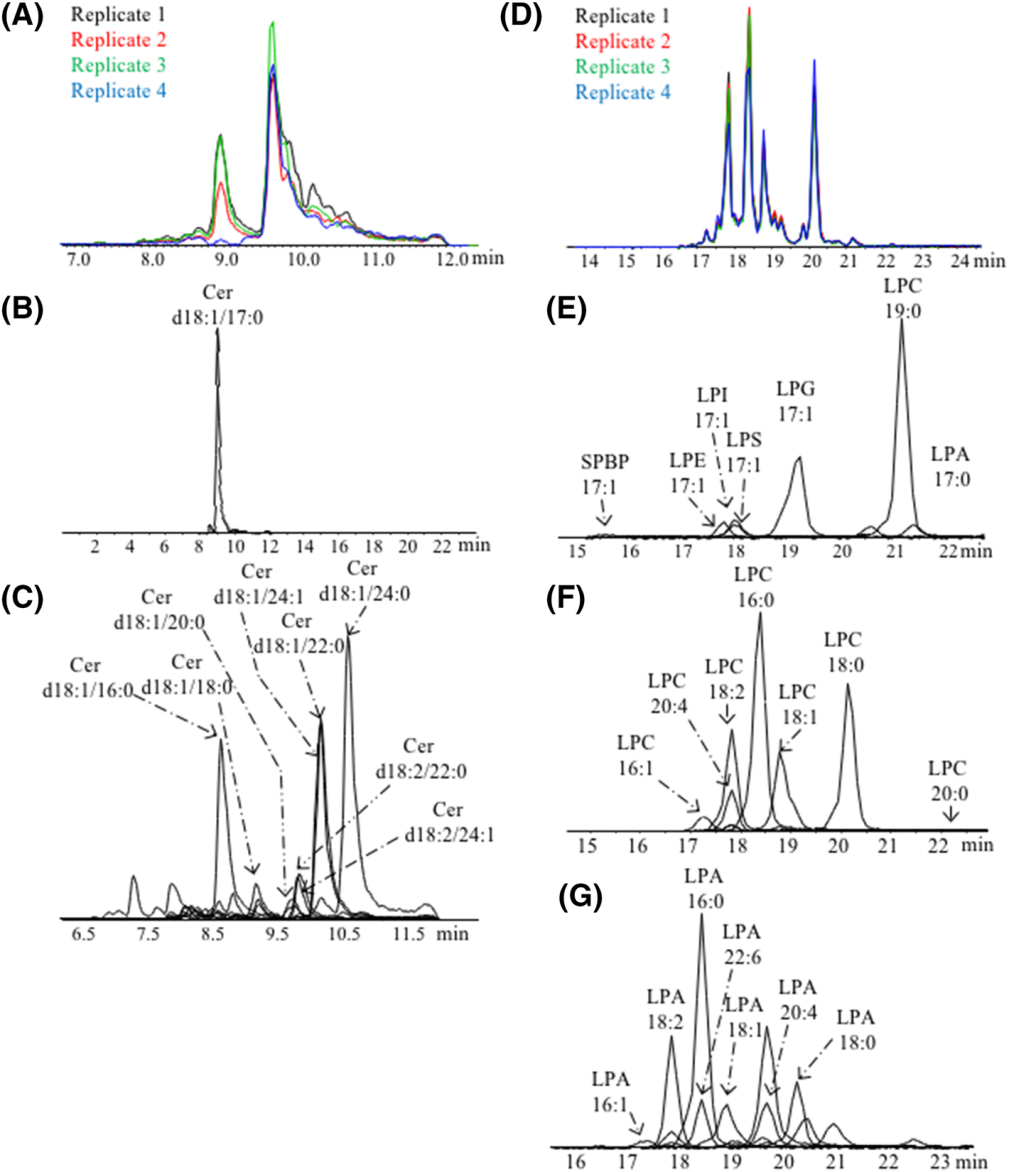
LC–MS/MS (liquid chromatography–tandem mass spectrometry) analysis of SRM 1950. A, Total ion count of Cer (ceramide) molecular species with sphingosine base d18:1 and sphingoid base 18:2. B, Extracted ion count of Cer (d18:1/17:0) internal standard spiked to each replicate. C, Extracted ion count of Cer molecular species. D, Total ion count of monoacylphospholipids (lysolipids). E, Extracted ion count of lysolipids and sphingosine‐1‐phosphate (SPBP) internal standards. F, Extracted ion count of lysophosphocholine (LPC) molecular species. G, Extracted ion count of each lysophosphatidic acid (LPA) molecular species [Color figure can be viewed at wileyonlinelibrary.com]

Complementing this comprehensive profiling of SRM 1950, analysis of SPs shows similar Cer molecular species as reported in the consensus^39^ (Table S4 [supporting information]). The TIC obtained for the Cer in all the four replicates for SRM 1950 is presented in Figure 3A, showing good repeatability within the method. IS Cer (d18:1/17:0) eluted at 8.99 min (Figure 3B). Cer molecular species with shorter acyl chains eluted before the IS, whereas Cer with longer acyl chains eluted after the RT of the IS, in line with separation by the acyl chain and number of double bonds in the method presented here. In addition to Cer with sphingoid base d18:1, Cer with sphingoid bases 18:2 and 18:0 were observed (Figure 3C; Table S4 [supporting information]), which were not reported using high‐throughput methods.^38^, ^39^ Other SPs measured using this comprehensive method include sphingosine (SPB 18:1) and dihydrosphingosine/sphinganine (dhSPB 18:0), which were not detected by the harmonised consensus.^39^

Interestingly, the lipid subclass SPBP was separated with the LC conditions used for lysolipid detection (Figures 3D and 3E). The lysolipid LC method enabled the separation of LPA, LPS, LPI, LPG, LPE and LPC molecular species, as shown by the separation of the ISs spiked into SRM 1950. Figures 3F and 3G show the separation of the different LPC and LPA molecular species detected in SRM 1950. It can be observed that the elution of LPA 18:1 does not overlap that of LPC 18:1, which is necessary as LPC decomposes in the source to form LPA. Other lysolipid subclasses and molecular species are reported in Table S5 (supporting information). In previous publications using high‐throughput methods with Folch extraction, it had only been possible to report LPC, O‐LPC/P‐LPC, P‐LPE, LPS, LPG and O‐LPA/P‐LPA species and six to eight molecular species of LPE but no LPA.^8^, ^9^, ^41^, ^42^ Additional LPE species were not detected by more than five laboratories, as reported by Bowden et al.^39^ Lysolipid results, including all the LPE species reported by Bowden and co‐authors, showed a similar abundance distribution to the distribution obtained using this comprehensive method (Figure S9 [supporting information]).

Cumulatively, 1364 lipid molecular species were identified across the four SRM 1950 replicates (Table S5 [supporting information]). Here, only even‐chain molecular species are included as fatty acids obtained from either endogenous fatty acid biosynthesis or dietary uptake are lengthened in two‐carbon increments from the carboxylic acid end.^43^ Therefore, comparison of the lipid profiles obtained for SRM 1950 without the odd species is considered in the summary of results presented in Table 3. In addition, previously reported data as the sum composition of fatty‐acyl‐chain carbon atoms and double bonds of GP and GL are presented in Table 3. Table 3 includes lipid categories and the number of molecular species quantified using these comprehensive methods (results reported in column 1) and nine publications that reported the lipid profile of SRM 1950. Some results were obtained using class‐specific methods reported by a single laboratory using multiple extraction methods and derivatisation for specific lipid subclasses like Quehenberger et al.^40^ Results from Huynh et al are not included as their results include acyl‐chain composition of GP and GL.^42^

**TABLE 3:** Comparison of the number of molecular species identified using the method presented here and previous publications for SRM 1950

Lipid category	Number of molecular species									
	1	2^40^	3^39^	4^44^	5^38^	6^45^	7^9^	8^46^	9^47^	10^48^
Glycerolipids	95	73	39	36	0	89	122	0	2	95
TG	75	18	34	17	–	69	97	–	–	80
DG	9	55	5	19	–	17	24	–	2	15
O‐DG	1	–	–	–	–	–	1	–	–	–
O‐TG	10		0	–	–	3	–	–	–	–
Glycerophospholipids	192	162	109	42	110	126	198	116	18	76
PC	27	24	24	7	19	54	65	31	4	27
PE	12	27	15	4	11	6	12	17	3	6
PI	17	19	13	2	16	–	6	–	4	–
PA	Δ	15	–	–	5	–	–	–	–	–
PG	7	16	1	2	8	–	–	–	2	–
*N*‐Acyl‐PS	–	2	–	–	–	–	–	–	–	–
PS	–	20	–	1	3	–	–	–	–	–
CL	Δ	–	–	–	–	–	–		–	–
LPC	23	9	22	19	12	26	31	17	4	11
LPE	11	7	6	2	14	9	–	7	1	3
LPS	5	–	–	–	–	–	–	–	–	–
LPI	6	–	–	–	–	–	–	–	–	–
LPG	12	–	–	–	4	–	–	–	–	–
LPA	11	–	–	–	18	–	–	–	–	–
O‐LPA/P‐LPA	4	–	–	–	–	–	–	–	–	–
O‐LPE/P‐LPE	5	–	–	–	–	–	–	–	–	–
O‐LPC/P‐LPC	9	3	–	–	–	7	–	–	–	–
O‐PE/P‐PE	20	13	13	2	–	5	15	15	–	9
O‐PC/P‐PC	23	7	15	3	–	19	69	29	–	20
Glycerophospholipids	95	73	39	36	0	89	122	0	2	95
SM (d18:1)	15	25	17	8	–	52	39	24	–	20
Cer (d18:1)	9	41	7	7	–	13	7	–	2	6
dhCer (d18:0)	5	–	–	–	–	–	–	–	–	–
dhSM (d18:0)	3	–	2	–	–	–	–	–	–	–
SPB (d18:1)	1	–	–	–	–	–	–	–	–	–
dhSPB (d18:0)	1	–	–	–	–	–	–	–	–	–
SPBP (d18:1)	1	1	–	–	–	–	–	–	–	–
SPBP (d18:0)	1	1	–	–	–	–	–	–	–	–
SPBP (d18:2)	1	–	–	–	–	–	–	–	–	–
Hex‐Cer	*	35	4	4	–	6	6	–	–	3
Sphingolipids	37	103	30	19	0	71	52	24	2	29
ST	1	–	1	–	–	–	–	–	–	–
CE	13	–	13	4	–	8	–	–	–	14
**Sterols**	**14**		**14**	**4**		**8**				**14**
Total	**338**	**338**	**192**	**101**	**110**	**294**	**372**	**140**	**22**	**214**

*Notes*: The current method (number 1) is compared to previous publications (numbers 2–10), in which the superscript numbers correspond to each reference. *Not measured; −not reported; ^Δ^measured but not detectable in human plasma.

Abbreviations: Cer, ceramide; CL, cardiolipin; DG, diacylglycerol; dhSPBP, dihydrosphigosine‐1‐phosphate; dhSM, dihydrosphingomyelin; LPA, lysophosphatidic acid; LPE, lysophosphatidylethanolamine; LPI, lysophosphoinositol; LPS, lysophosphoserine; O‐DG, alkyl‐diacylglycerol; O‐LPA, alkyl‐lysophosphatidic acid; O‐LPC, alkyl‐lysophosphatidylcholine; O‐LPE, alkyl‐lysophosphatidylethanolamine; O‐PC, alkyl‐acylglycerophosphocholine; O‐PE, alkyl‐acylglycerophosphoethanolamine; O‐TG, alkyl‐triacylglycerol; PA, phosphatidic acid; PC, phosphatidylcholine; PE, phosphatidylethanolamine; PG, phosphatidylglycerol; PI, phosphatidylinositol; P‐LPA, alkenyl‐lysophosphatidic acid; P‐LPC, alkenyl‐lysophosphatidylcholine; P‐LPE, alkenyl‐lysophosphatidylethanolamine; P‐PC, alkenyl‐acylglycerophosphocholine; P‐PE, alkenyl‐acylglycerophosphoethanolamine; PS, phosphatidylserine; SM, sphingomyelin; SPBP, sphingosine‐1‐phosphate; TG, triacylglycerol.

Table 3 presents lipid subclasses LPS, LPI, O‐LPA/P‐LPA, O‐LPE/P‐LPE, dhCer (d18:0), SPB (d18:1), dhSPB (d18:0) and SPBP (d18:2). These are very‐low‐abundance lipid subclasses (or molecular species in the case of SPs), which can be detected using only the comprehensive method presented here. The results presented in columns 2–10 in Table 3 do not include the results for the 10 lipid subclasses mentioned earlier. In addition, 338 molecular species were detected using the comprehensive chromatographic methods described here. Interestingly, the total number of molecular species detected using our comprehensive method matches the total number of molecular species obtained by Quehenberger et al,^40^ where a variety of chromatographic methods for the analysis of different lipid subclasses were used.

These results highlight the need for standardisation in the lipidomics field,^49^ where comprehensive analyses by parallel and serial use of analytical techniques are suited to reflect lipid subclass diversity.^34^ The need for standardised methodologies and workflows for semi‐quantitative approaches might explain the inconsistency of our results with those previously reported for these samples as stated earlier. Nonetheless, our comprehensive approach is shown to have potential for comprehensive lipidomics profiling studies. There is still a general limitation to assess both acyl‐chain position (*sn*‐1 or *sn*‐2) for GP and DG and double‐bond position within the same sample in a single chromatographic run. Monitoring of acyl chains can be implemented for positively charged GP and GL in any triple quadrupole instrument using the LC methods presented here. However, multiple column injections would be necessary as each acyl‐chain fragment needs to be monitored for the same precursor. Therefore, the MRM list becomes very extensive for all the molecular species with two, three or four acyl chains (PC, TG and CL, respectively). Even though the lists can be long, the minimum number of points recommended to define a peak is 10, and this number is calculated by the scan time divided by the peak width.

The methods presented here enable the identification and relative quantification of a number of low‐ and very‐low‐abundance signalling lipids, which are of interest due to their role in the development of certain diseases (10.1161/ATVBAHA.120.305565).^50^ Furthermore, the identification of the majority of lipid subclasses involved in different metabolic routes like *de novo* lipogenesis and SPs also enables the building of lipid metabolic pathways, using tools such as BioPAN (10.12688/f1000research.28022.2)^51^(freely accessible in the LIPID MAPS website at https://www.lipidmaps.org/biopan/). As lipids are substrates, products and intermediates in these metabolic reactions and a lack of any substrates and/or products would lead to an incomplete pathway. The methods presented here have the potential to be used for a variety of matrices, such as tissues, cells and other biofluids; however, each matrix must be validated. Although considerable time is required for running the LC–MS methods, the minimal sample manipulation avoids the need for derivatisation for lipids such as SPBP and dhSPBP.

## 4 CONCLUSIONS

This work developed a workflow for lipid profiling using three different extraction methods and four different LC–MS and LC–MS/MS methods. The comprehensive methods described here successfully separated both PA from PS and PC, and LPA from LPA and LPC, enabling confident identification of several lipid molecular species. The results of GLs, GPs, sterols and SPs are in good agreement with previously reported results in the NIST SRM 1950 sample by other laboratories. Ten lipids subclasses LPS, LPI, O‐LPA/P‐LPA, O‐LPE/P‐LPE, dhCer (d18:0), SPB (d18:1), dhSPB (d18:0) and SPBP (d18:2) have been detected using this comprehensive method.

### PEER REVIEW

The peer review history for this article is available at https://publons.com/publon/10.1002/rcm.9472.

## Supporting information


**FIGURE S1** Comparison of ionisation efficiency. Extracted internal standard total and extracted ion chromatogram with and without the post‐column addition of ethylamine for the neutral lipids DG, TG and CE
**FIGURE S2** Comparison of the residual sodium adducts signal. Extracted internal standard total and extracted ion chromatogram with and without addition of ethylamine for the neutral lipids DG, TG and CE
**FIGURE S3** Glycerophospholipids and sphingomyelin LC method
**FIGURE S4** Glycerolipids and sterols LC method
**FIGURE S5** LC method lysolipids and SPBP
**FIGURE S6** LC method ceramides and SPB
**FIGURE S7** Calibration curves of selected internal standards spiked into SRM 1950 plasma matrix for validation of linearity of HPLC/ESI‐MS methods
**FIGURE S8** Sphingomyelin (SM) profile obtained in human plasma SRM 1950: SM comprehensive are the results produced using the GP method described here. SM harmonised are the results obtained by Bowden et al
**FIGURE S9** Phosphatidylinositol (PI) profile obtained in human plasma SRM 1950: PI comprehensive are the results produced using the GP method described here. PI harmonised are the results obtained by Bowden et al
**FIGURE S10** Phosphatidylethanolamine (PE) profile obtained in human plasma SRM 1950: PE comprehensive are the results produced using the GP method described here. PE harmonised are the results obtained by Bowden et al
**FIGURE S11** Triacylglycerol (TG) profile obtained in human plasma SRM 1950: TG comprehensive are the results produced using the GP method described here. TG harmonised are the results obtained by Bowden et al
**FIGURE S12** Cholesteryl ester (CE) profile obtained in human plasma SRM 1950: CE comprehensive are the results produced using the GL method described here. CE harmonised are the results obtained by Bowden et al
**FIGURE S13** LPE profile obtained in human plasma SRM 1950: LPE comprehensive are the results produced using the GP method described here. LPE harmonised are the results obtained by Bowden et al
**TABLE S1** Comparison of peak areas obtained with the formation of each cation adduct. Extracted internal standard with and without the post‐column addition of ethylamine for the neutral lipids DG, TG and CE. Na represents each internal standard with the formation of an adduct with the residual Na^+^[M + Na]^+^; _NH_4_ represents each internal standard with the addition of ammonium for ionisation [M + NH_4_]^+^, and _C_2_H_7_N represents each internal standard with the addition of ethylamine for ionisation [M + C_2_H_7_N^+^]
**TABLE S2** List of internal standards used in this study. The standards were purchased from Avanti (Alabaster, AL, USA)
**TABLE S3** MRM transitions, dwell time and collision energies used for lysolipids and *SPBP* LC–MS/MS analysis
**TABLE S4** MRM transitions, dwell time and collision energies used for ceramides, dihydroceramides and sphingosine LC–MS/MS analysis
**TABLE S5** Lipidome in SRM 1950

## Data Availability

Data described in the manuscript can be shared upon request (andrea.lopez@babraham.ac.uk).
