# Supplementary material for: Comprehensive lipidome of human plasma using minimal sample manipulation by liquid chromatography coupled with mass spectrometry
Source: Rapid Commun Mass Spectrom. 2023 Feb 9;39(Suppl 1):e9472. doi: 10.1002/rcm.9472 (PMC12062770; doi:10.1002/rcm.9472)
Supplement: Supplementary file 1 — FIGURE S1 Comparison of ionisation efficiency. Extracted internal standard total and extracted ion chromatogram with and without the post‐column addition of ethylamine for the neutral lipids DG, TG and CE FIGURE S2 Comparison of the residual sodium adducts signal. Extracted internal standard total and extracted ion chromatogram with and without addition of ethylamine for the neutral lipids DG, TG and CE FIGURE S3 Glycerophospholipids and sphingomyelin LC method FIGURE S4 Glycerolipids and sterols LC method FIGURE S5 LC method lysolipids and SPBP FIGURE S6 LC method ceramides and SPB FIGURE S7 Calibration curves of selected internal standards spiked into SRM 1950 plasma matrix for validation of linearity of HPLC/ESI‐MS methods FIGURE S8 Sphingomyelin (SM) profile obtained in human plasma SRM 1950: SM comprehensive are the results produced using the GP method described here. SM harmonised are the results obtained by Bowden et al FIGURE S9 Phosphatidylinositol (PI) profile obtained in human plasma SRM 1950: PI comprehensive are the results produced using the GP method described here. PI harmonised are the results obtained by Bowden et al FIGURE S10 Phosphatidylethanolamine (PE) profile obtained in human plasma SRM 1950: PE comprehensive are the results produced using the GP method described here. PE harmonised are the results obtained by Bowden et al FIGURE S11 Triacylglycerol (TG) profile obtained in human plasma SRM 1950: TG comprehensive are the results produced using the GP method described here. TG harmonised are the results obtained by Bowden et al FIGURE S12 Cholesteryl ester (CE) profile obtained in human plasma SRM 1950: CE comprehensive are the results produced using the GL method described here. CE harmonised are the results obtained by Bowden et al FIGURE S13 LPE profile obtained in human plasma SRM 1950: LPE comprehensive are the results produced using the GP method described here. LPE harmonised are the results obtained by Bowden et al TABLE S1 Comparison [file RCM-39-e9472-s001.doc]

***Supporting Information***

# Comprehensive lipidome of human plasma using minimal sample manipulation by liquid chromatography coupled with mass spectrometry.

Bebiana C. Sousa^1^, Zulema Gonzalez Klein^1,2,3^, Diane Taylor^1^, Greg West^1^, Aveline Neo Huipeng^1^, Michael J.O. Wakelam^1^, Andrea F Lopez-Clavijo^1*^

*^1^ Lipidomics facility, Babraham Institute, Babraham Research Campus, Cambridge, CB22 3AT, United Kingdom.* ^2^ *Centro de Biotecnología y Genómica de Plantas (CBGP), Instituto Nacional de Investigación y Tecnología Agraria y Alimentaria (INIA), Universidad Politécnica de Madrid (UPM), Madrid, Spain.* ^3^ *Departamento de Biotecnología-Biología Vegetal, Escuela Técnica Superior de Ingeniería Agronómica, Alimentaria y de Biosistemas, Universidad Politécnica de Madrid (UPM), Madrid, Spain*

**Table of contents**

Figure S-1. Comparison of the ionisation efficiency…………………………………………….3

Figure S-2. Comparison of the residual sodium adducts signal…………………………….........4

Table S-1. Comparison of peak areas obtained with the formation of each cation adduct………5

[Table S-2. List of internal standards used in this study…………………………………………..6](#__RefHeading___Toc117253031)

[Section S-1. Lipid Extraction….………………………………………………………………….7](#__RefHeading___Toc117253032)

[*S1.1 Folch Extraction* 7](#__RefHeading___Toc117253033)

[*S1.2 Folch-Butanol Extraction* 7](#__RefHeading___Toc117253034)

[Section S-2 - Liquid chromatography coupled to mass spectrometry (LC-MS) analysis………..9](#__RefHeading___Toc117253035)

[*S-2.1 Glycerophospholipids and Sphingomyelin* 9](#__RefHeading___Toc117253036)

[Figure S-3. Glycerophospholipids and Sphingomyelin LC method 9](#__RefHeading___Toc117253037)

[*S-2.2 Glycerolipids and Sterols* 9](#__RefHeading___Toc117253038)

[Figure S-4. Glycerolipids and sterols LC method 10](#__RefHeading___Toc117253039)

[*S-2.3 Lysolipids and SPBP* 11](#__RefHeading___Toc117253040)

[Figure S5. LC method lysolipids and SPBP 11](#__RefHeading___Toc117253041)

[Table S-3. MRM transitions, dwell time and collision energies used for lysolipids and *SPBP* LC-MS/MS analysis. 11](#__RefHeading___Toc117253042)

[*S2.4 Ceramides and Sphingosine* 14](#__RefHeading___Toc117253043)

[Figure S-6. LC method ceramides and SPB 14](#__RefHeading___Toc117253044)

[Table S-4. MRM transitions, dwell time and collision energies used for ceramides, dihydroceramides, and sphingosine LC-MS/MS analysis 15](#__RefHeading___Toc117253045)

[Figure S-7. Calibration curves of selected internal standards spiked into SRM1950 plasma matrix for validation of linearity of HPLC/ESI-MS methods………………………………………………16](#__RefHeading___Toc117253046)

[Table S-5. Lipidome in SRM 1950………………………………………………………………17](#__RefHeading___Toc117253047)

[Figure S-8. Sphingomyelin (SM) profile obtained in human plasma SRM 1950………………..25](#__RefHeading___Toc117253048)

[Figure S-9. Phosphatidylinositol (PI) profile obtained in human plasma SRM 1950……………26](#__RefHeading___Toc117253049)

[Figure S-10. Phosphatidylethanolamine (PE) profile obtained in human plasma SRM 1950……27](#__RefHeading___Toc117253050)

[Figure S-11. Triacylglycerols (TG) profile obtained in human plasma SRM 1950………………28](#__RefHeading___Toc117253051)

[Figure S-12. Cholesteryl ester (CE) profile obtained in human plasma SRM 1950……………...29](#__RefHeading___Toc117253052)

[Figure S-13. LPE profile obtained in human plasma SRM 1950………………………………....30](#__RefHeading___Toc117253053)

**F**igure S-1. Comparison of the ionisation efficiency. Extracted internal standard total and extracted ion chromatogram with and without the post column addition of ethylamine for the neutral lipids DG, TG, and CE.

| a. |  |  |
| --- | --- | --- |
| b |
| c |
| d |
| STDx no ethylamine addition [M + NH_4_]^+^   1. TIC 2. EIX of Internal standard TG 51:3 ammonium adduct m/z = 860.7701 3. EIX of Internal standard DG 35:1 ammonium adduct m/z = 626.5723 4. EIX of Internal standard CE 17:0 ammonium adduct m/z = 656.6345 | | STDx ethylamine addition [M + C_2_H_7_N]^+^   1. TIC 2. EIX of Internal standard TG 51:3 ethylamine adduct m/z = 888.8015 3. EIX of Internal standard DG 35:1 ethylamine adduct m/z = 654.6031 4. EIX of Internal standard CE 17:0 ethylamine adduct m/z = 684.6653 |

Figure S-2. Comparison of the residual sodium adducts signal. Extracted internal standard total and extracted ion chromatogram with and without addition of ethylamine for the neutral lipids DG, TG, and CE.

| a. |  |  |
| --- | --- | --- |
| b |
| c |
| d |
| STDx no ethylamine addition [M + Na]^+^   1. TIC 2. EIX of Internal standard TG 51:3 sodium adduct m/z = 865.7256 3. EIX of Internal standard DG 35:1 sodium adduct m/z = 631.5272 4. EIX of Internal standard CE 17:0 sodium adduct m/z = 661.5894 | | STDx ethylamine addition [M + Na]^+^   1. TIC 2. EIX of Internal standard TG 51:3 sodium adduct m/z = 865.7256 3. EIX of Internal standard DG 35:1 sodium adduct m/z = 631.5272 4. EIX of Internal standard CE 17:0 sodium adduct m/z = 631.5272 |

Table S-1. Comparison of peak areas obtained with the formation of each cation adduct. Extracted internal standard with and without the post column addition of ethylamine for the neutral lipids DG, TG, and CE. _Na represent each internal standard with the formation of an adduct with the residual Na^+^ [M + Na]^+^; _NH_4_ represent each internal standard with the addition of ammonium for ionisation [M + NH_4_]^+^, and _C_2_H_7_N represent each internal standard with the addition of ethylamine for ionisation [M + C_2_H_7_N^+^].

| **No post-column addition of ethylamine** | | | **Post-column addition of ethylamine** | | | **Expected retention time of the**  **Internal standard** |
| --- | --- | --- | --- | --- | --- | --- |
| **TG species** | **Area** | ***m/z*** | **TG species** | **Area** | **m/z** |
| **TG51:3_19.17_Na** | 41080616 | 865.7256 | **IS51:3_19.26_Na** | 3296043 | 865.7256 | TG 53:1 = 19.37 min |
| **TG51:3_19.17_NH4** | 714487872 | 860.7701 | **IS51:3_19.26_C2H7N** | 5.11E+08 | 888.8015 |
| **DG species** | **Area** | ***m/z*** | **DG species** | **Area** | **m/z** | d5DG 34:0 = 13.31 min  DG 35:1 = 13.37 min |
| **d5DG 34:0_3.24_Na** | 86486344 | 614.4881 | **d5DG 34:0_3.27_Na** | 17503790 | 614.4881 |
| **d5DG 34:0_9.61_NH4** | 2639237 | 609.5332 | **d5DG 34:0_9.74_Na** | 5438693 | 614.4881 |
| **d5DG 34:0_10.36_NH4** | 3320400 | 609.5332 | **d5DG 34:0_10.91_Na** | 2968138 | 614.4881 |
| **d5DG 34:0_13.31_NH4** | 2396132 | 609.5332 | **d5DG 34:0_13.31_Na** | 626496.1 | 614.4881 |
| **DG35:1_13.44_Na** | 67336502 | 631.5272 | **d5DG34:0_13.31_C2H7N** | 26599731 | 637.564 |
| **DG35:1_13.46_NH4** | 2E+08 | 626.5723 | **DG 35:1_13.50_Na** | 15460241 | 631.5272 |
|  |  |  | **DG 35:1_13.50_C2H7N** | 2.37E+08 | 654.6031 |
| **CE species** | **Area** | ***m/z*** | **CE species** | **Area** | **m/z** | CE 17:0 = 21.52 min |
| **CE 17:0_21.87_Na** | 1966289 | 661.5894 |  |  |  |
| **CE 17:0_21.87_NH4** | 24215565 | 656.6345 | **CE 17:0_22.00_C2H7N** | 14617864 | 684.6653 |

Table S-2. List of internal standards used in this study**.** The standards were purchased from Avanti (Alabaster, AL, USA).

| **Lipid Category** | **Lipid Classes/Subclasses** | **Shorthand notation** | **Molecular species** | | **Conc**  **ng/µL** |
| --- | --- | --- | --- | --- | --- |
|  |  |  | Sum composition | Acyl chain composition |  |
| Glycerophospholipids (GP) | Diacylglycerophosphocholine | PC | 35:1 | 17:0/18:1 | 1 |
|  | Diacylglycerophosphate | PA | 35:1 | 17:0/18:1 | 3 |
|  | Diacylglycerophosphoethanolamine | PE | 35:1 | 17:0/18:1 | 1 |
|  | Diacylglycerophosphoserine | PS | 35:1 | 17:0/18:1 | 1 |
|  | Diacylglycerophosphoinositol | PI | 37:4 | 17:0/20:4 | 1 |
|  | Diacylglycerophosphoglycerol | PG | 35:1 | 17:0/18:1 | 1 |
|  | Diacylglycerophosphoglycerophosphodiradylglycerol (Cardiolipin) | CL | 56:0 | 14:0/14:0/14:0/14:0 | 3 |
|  | Monoacylglycerophosphate | LPA | 17:0 | | 0.1 |
|  | Monoacylglycerophosphocholine | LPC | 19:0 | | 0.1 |
|  | Monoacylglycerophosphoethanolamine | LPE | 17:1 | | 0.1 |
|  | Monoacylglycerophosphoglycerol | LPG | 17:1 | | 0.1 |
|  | Monoacylglycerophosphoinositol | LPI | 17:1 | | 0.1 |
|  | Monoacylglycerophosphoserine | LPS | 17:1 | | 0.1 |
| Glycerolipids (GL) | Diacylglycerol | DG | 35:1 | 17:0/18:1 | 1 |
|  | Triacylglycerol | TG | 68:4 | 17:1/17:1/17:1/17:1 | 1 |
|  | Monoacylglycerol | MG | 17:0 | | 1 |
| Sphingolipids (SP) | Ceramide phosphocholines (sphingomyelins) | SM | 35:1 | d18:1:17:0 | 1 |
|  | N-acylsphingosines (ceramides) | Cer | 35:1 | d18:1/17:0 | 1 |
|  | Sphingoid base | SPB | 18:1 | | 1 |
|  | Sphingoid base 1-phosphate | SPBP | 17:1 | | 0.1 |
| Sterol Lipids (ST) | Cholesterol | ST | D7 | | 4.8 |
|  | Sterol esters | CE | 17:0 | | 1 |

# Section S-1 - Lipid Extraction

## *S1.1 Folch Extraction*

To a 4 mL silanized tube, 800 μL of methanol were added followed by the addition of 10 μL of a mixture of standards at a concentration of 1ng/μL, as listed above. Then, 10 μL of plasma and 780 μL of Milli-Q water were added, followed by the addition of 1600 μL of chloroform to a final chloroform/methanol/water ratio of 2:1:1 (v/v). The samples were then vortexed for 5 seconds and centrifuged at 3200 g for 5 min at room temperature to obtain two-phases: an aqueous upper phase and an organic lower phase. The lipid extract present in the organic phase was transferred to a new clean 4 mL silanized tube. To achieve higher recovery, 1.6 mL of chloroform were added to the aqueous phase, which was then vortexed for 5 seconds and centrifuged (3200g, 5 min, RT). The organic phase was recovered to the same tube as before and the combined organic phases were dried in a speedvac (Savant SP131DDA, Thermo Scientific, Runcorn, UK) under 2.00 torr pressure and no temperature for 45 minutes. After drying, the lipid extracts were re-suspended in 50 μL of chloroform/methanol 1:1 (v/v) and transferred to LC-MS vials (Thermo Scientific, Runcorn, UK). Four replicates were prepared and analysed to verify the reproducibility of the results.

## *S1.2 Folch-Butanol Extraction*

Firstly, to a 4 mL silanized tube, 1500 μL of chloroform/methanol 2:1 (v/v) were added followed by 10 μL of a mixture of standards at a concentration of 0.1ng/ μL. Then, 20 μL of plasma and 480 μL of Milli-Q water were added before vortexing for 15 seconds and centrifuging at 3200 g rpm for 5 minutes. A clear phase separation was obtained, and the lower organic phase was transferred to a clean 4 mL silanized tube and then dried in a speedvac under 2.00 torr pressure and no temperature for 45 minutes. The remaining upper phase was extracted with 500 μL of Milli-Q water and 1000 μL of butanol, then vortexed for 15 seconds and centrifuged (3200 g, 5 min, RT). The butanol upper phase was transferred to a clean 5 mL Eppendorf tube. 500 μL of butanol were added to the remaining lower phase for re-extraction, followed by another cycle of vortexing and centrifugation. The new butanol upper phase was collected and combined with the previous butanol phase in the same 5 mL Eppendorf tube. A solution of butanol/water 1:1 (v/v) was prepared prior to extraction creating two layers, a lower butanol-saturated water, and an upper water-saturated butanol layer. 1000 μL of butanol-saturated water were added to the combined butanol layers, before vortexing for 15 seconds and centrifugation (3200 g, 5 min, RT). The upper phase was transferred to a clean 2.5 mL Eppendorf tube, and 500 μL of water-saturated butanol were added to the remaining lower phase, before it was vortexed for 15 seconds and centrifuged (3200 g, 5 min, RT). The upper phase was collected, combined with the previous butanol phase in the 2.5 mL Eppendorf tube and dried in a speedvac under 2.00 torr pressure and no temperature for 150 minutes. After drying, the lipid extracts were re-suspended in 50 μL of acidified methanol and transferred to LC-MS vials containing 100 μL inserts for higher recovery. Acidified methanol was prepared by the addition of 500 μL of 1M ammonium formate and 580 μL formic acid to 49.5 mL of methanol. Four replicates were prepared and analysed to verify the reproducibility of the results.

# Section S-2 - Liquid chromatography coupled to mass spectrometry (LC-MS) analysis

## *S-2.1 Glycerophospholipids and Sphingomyelin*

The solvent gradient was programmed as follows (**Figure S-3**): 10% B was held isocratic for 5 minutes and increased to 30% at minute 6.00. A 10% increase was achieved in the next minute followed by an increase of 5% in 0.5 minutes. Then the gradient was increased to 50% at minute 8.00. Later, 10% was increased in 1.5 minutes and 5% more in 0.5 minutes. At minute 13.00, the gradient was set up at 70%. It was then increased reaching 90% at 21.0 minutes and 95% at minute 23.0 where it was held for 1 minute before it was switched off two minutes later. Solvent C flow was set to 0.6 mL/min, started at minute 27.0, and left running for 5 minutes before it was switched off. Then at 32 minutes pump B was switched on at 10% and was increased to 90% B two minutes before returning to the initial condition of 10% B one minute later. The oven temperature was set to 40 °C. The flow rate was set to 0.4 mL/min and it was ionised in negative ion mode under the HESI conditions: electrospray voltage, 3.10 kV; capillary temperature, 375 °C; heater temperature 375 °C; sheath gas rate 35 U; aux gas rate 15 U; and S-Lens RF level 60.0%. Data acquisition was carried out on an Xcalibur data system (version 3.0.63.3). Lipid Data Analyser (LDA, Graz University of Technology, version 2.6.3) was used to assign each *m/z* with an error less than 5 ppm using deprotonated [M-H]- specie for PI, PG, PA, PS, PE, O-PE, and CL. The formate adduct [M+ HCOO]^-^ was used to identify PC, SM, dhSM, O-PC, and P-PC.


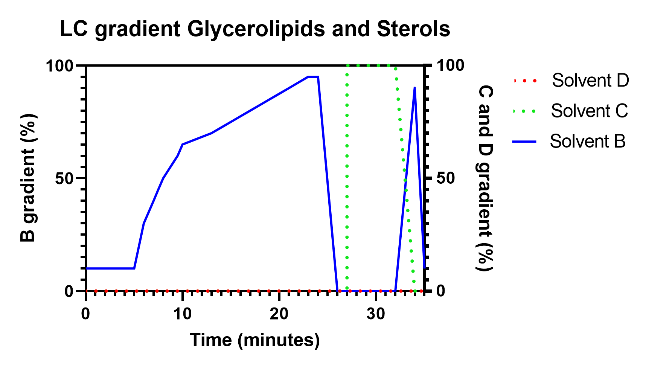


**Figure S-3. Glycerophospholipids and Sphingomyelin LC method**

## *S-2.2 Glycerolipids and Sterols*

The solvent gradient was programmed as follows (**Figure S-4**): it started with 60% B and was held isocratic for 5 minutes. Linearly, it increased to 70% B over 2 minutes, then to 90% B over 13 minutes and then to 99% B over a further 2 minutes. The flow was held at 99% B for 2 minutes before it was decreased to 0%, during which time Pump D flow was set to 0.15 mL/min and held for 5 minutes to clean the column. The flow was then returned linearly to 60% B over 9 minutes and was held at 60% B for 3 minutes for column equilibration. Post column addition (Pump C) was active throughout the whole run at a flow rate of 0.15 mL/min, except between the minutes 9 and 11 when this pump was off for the elution of ST. The oven temperature was set to 40 °C. The flow rate was set to 0.15 mL/min and it was ionised in positive ion mode under the HESI conditions: electrospray voltage, 3.50 kV; capillary temperature, 325 °C; heater temperature 275 °C; sheath gas rate 25 U; aux gas rate 10 U; and S-Lens RF level 60.0%. Data acquisition was carried out on an Xcalibur data system (version 3.0.63.3). Lipid Data Analyser was used to assign each *m/z* with an error of less than 5 ppm, using protonated [M-H_2_O]^+^ specie for ST. The ethylamine adduct [M+C_2_H_7_N]^+^ was used to identify DG, TG, O-DG, O-TG, and CE.


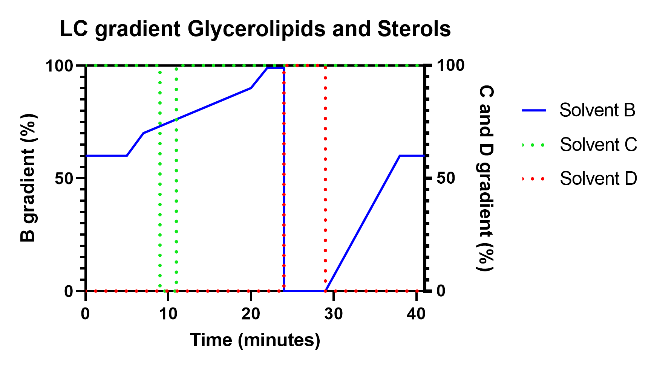


### **Figure S-4. Glycerolipids and sterols LC method**

## *S-2.3 Lysolipids and SPBP*


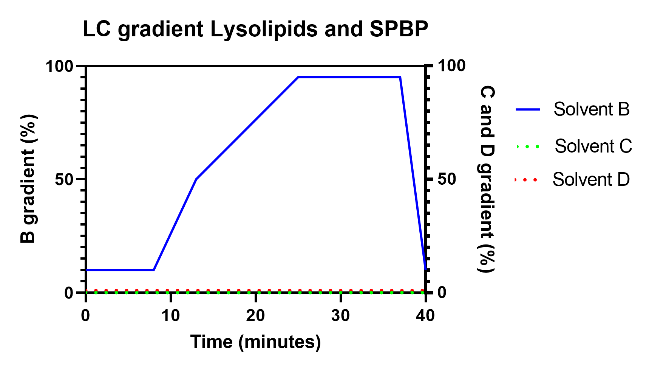
The solvent gradient was programmed as follows (**Figure S-5**): it started with 10% B and was held isocratic for 8 minutes. It increased linearly to 50% B over 5 minutes, and then to 95% B over 12 minutes after which it was held for another 12 minutes isocratic, before returning to 10% B over 3 minutes for column equilibration. The oven temperature was set to 40 °C. The flow rate was set to 0.2 mL/min and it was redirected to a QTRAP mass spectrometer. The mass spectrometer was operated in negative-ion mode and the ESI conditions were used as follows: curtain gas, 20.0; collision gas, medium; ion spray voltage, -4500 V; temperature, 400 ⁰C; ion source gas 1, 40.0; ion source gas 2, 30.0; declustering potential, -200; entrance potential, -10.0; and collision cell exit potential, -13.0. MS/MS analysis was carried out in MRM scanning mode, using the chosen transitions to fix the precursor ion and product ion *m/z* values at the first and third quadrupole, respectively (**Table S-2**). The total scan time was 5.77 seconds, dwell time was 30.0 milliseconds, and the collision energy was -30 V at the second quadrupole for all transitions, except for the transitions corresponding to LPI species for which the collision energy was -45 V and SPBP for which collision energy was -35 V. Data acquisition was carried out on Analyst (version 1.7.1). SCIEX OS (Version 2.1.6.59781) was used for analysing the data.

### **Figure S5. LC method lysolipids and SPBP**

### **Table S-3. MRM transitions, dwell time and collision energies used for lysolipids and *SPBP* LC-MS/MS analysis.**

| **Lipid subclass** | **Molecular species** | **Q1 Mass (Da)** | **Q3 Mass (Da)** | **Dwell Time (msec)** | **CE (volts)** |
| --- | --- | --- | --- | --- | --- |
| LPA | 14:0 | 381.205 | 153.00 | 30.0 | -30.000 |
|  | 16:0 | 409.236 | 153.00 | 30.0 | -30.000 |
|  | 16:1 | 407.220 | 153.00 | 30.0 | -30.000 |
|  | 17:0 IS | 423.252 | 153.00 | 30.0 | -30.000 |
|  | 18:0 | 437.267 | 153.00 | 30.0 | -30.000 |
|  | 18:1 | 435.252 | 153.00 | 30.0 | -30.000 |
|  | 18:2 | 433.236 | 153.00 | 30.0 | -30.000 |
|  | 18:3 | 431.220 | 153.00 | 30.0 | -30.000 |
|  | 18:4 | 429.205 | 153.00 | 30.0 | -30.000 |
|  | 20:0 | 465.299 | 153.00 | 30.0 | -30.000 |
|  | 20:1 | 463.283 | 153.00 | 30.0 | -30.000 |
|  | 20:2 | 461.267 | 153.00 | 30.0 | -30.000 |
|  | 20:3 | 459.252 | 153.00 | 30.0 | -30.000 |
|  | 20:4 | 457.236 | 153.00 | 30.0 | -30.000 |
|  | 20:5 | 455.220 | 153.00 | 30.0 | -30.000 |
|  | 22:0 | 493.330 | 153.00 | 30.0 | -30.000 |
|  | 22:1 | 491.314 | 153.00 | 30.0 | -30.000 |
|  | 22:2 | 489.299 | 153.00 | 30.0 | -30.000 |
|  | 22:3 | 487.283 | 153.00 | 30.0 | -30.000 |
|  | 22:4 | 485.267 | 153.00 | 30.0 | -30.000 |
|  | 22:5 | 483.252 | 153.00 | 30.0 | -30.000 |
|  | 22:6 | 481.200 | 153.00 | 30.0 | -30.000 |
|  | 24:0 | 521.361 | 153.00 | 30.0 | -30.000 |
|  | 24:1 | 519.346 | 153.00 | 30.0 | -30.000 |
| O-LPA | 16:0 | 395.257 | 153.00 | 30.0 | -30.000 |
|  | 18:0 | 423.288 | 153.00 | 30.0 | -30.000 |
|  | 20:0 | 451.319 | 153.00 | 30.0 | -30.000 |
| P-LPA | 16:0 | 393.241 | 153.00 | 30.0 | -30.000 |
|  | 16:1 | 391.225 | 153.00 | 30.0 | -30.000 |
|  | 18:0 | 421.272 | 153.00 | 30.0 | -30.000 |
|  | 18:1 | 419.257 | 153.00 | 30.0 | -30.000 |
|  | 20:0 | 449.304 | 153.00 | 30.0 | -30.000 |
|  | 20:1 | 447.288 | 153.00 | 30.0 | -30.000 |
| LPC | 14:0 | 512.299 | 452.280 | 30.0 | -30.000 |
|  | 16:0 | 540.331 | 480.310 | 30.0 | -30.000 |
|  | 16:1 | 538.315 | 478.294 | 30.0 | -30.000 |
|  | 18:0 | 568.362 | 508.340 | 30.0 | -30.000 |
|  | 18:1 | 566.346 | 506.330 | 30.0 | -30.000 |
|  | 18:2 | 564.331 | 504.310 | 30.0 | -30.000 |
|  | 18:3 | 562.315 | 502.294 | 30.0 | -30.000 |
|  | 18:4 | 560.299 | 500.278 | 30.0 | -30.000 |
|  | 19:0 IS | 582.378 | 522.360 | 30.0 | -30.000 |
|  | 20:0 | 596.393 | 536.372 | 30.0 | -30.000 |
|  | 20:1 | 594.378 | 534.357 | 30.0 | -30.000 |
|  | 20:2 | 592.362 | 532.341 | 30.0 | -30.000 |
|  | 20:3 | 590.346 | 530.325 | 30.0 | -30.000 |
|  | 20:4 | 588.331 | 528.310 | 30.0 | -30.000 |
|  | 20:5 | 586.315 | 536.294 | 30.0 | -30.000 |
|  | 22:0 | 624.425 | 564.404 | 30.0 | -30.000 |
|  | 22:1 | 622.409 | 562.388 | 30.0 | -30.000 |
|  | 22:2 | 620.393 | 560.372 | 30.0 | -30.000 |
|  | 22:3 | 618.378 | 558.357 | 30.0 | -30.000 |
|  | 22:4 | 616.362 | 556.341 | 30.0 | -30.000 |
|  | 22:5 | 614.346 | 554.325 | 30.0 | -30.000 |
|  | 22:6 | 612.331 | 552.310 | 30.0 | -30.000 |
|  | 24:0 | 652.456 | 592.435 | 30.0 | -30.000 |
|  | 24:1 | 650.440 | 590.419 | 30.0 | -30.000 |
|  | 26:0 | 680.487 | 620.466 | 30.0 | -30.000 |
| O-LPC | 16:0 | 526.351 | 466.330 | 30.0 | -30.000 |
|  | 18:0 | 554.383 | 494.362 | 30.0 | -30.000 |
|  | 20:0 | 582.414 | 522.393 | 30.0 | -30.000 |
| P-LPC | 16:0 | 524.336 | 464.315 | 30.0 | -30.000 |
|  | 16:1 | 522.320 | 464.299 | 30.0 | -30.000 |
|  | 18:0 | 552.367 | 492.346 | 30.0 | -30.000 |
|  | 18:1 | 550.351 | 490.330 | 30.0 | -30.000 |
|  | 20:0 | 580.398 | 520.377 | 30.0 | -30.000 |
|  | 20:1 | 578.383 | 518.362 | 30.0 | -30.000 |
| LPE | 16:0 | 452.278 | 196.000 | 30.0 | -30.000 |
|  | 16:1 | 450.263 | 196.000 | 30.0 | -30.000 |
|  | 17:1 IS | 464.278 | 196.000 | 30.0 | -30.000 |
|  | 18:0 | 480.310 | 196.000 | 30.0 | -30.000 |
|  | 18:1 | 478.294 | 196.000 | 30.0 | -30.000 |
|  | 18:2 | 476.278 | 196.000 | 30.0 | -30.000 |
|  | 20:1 | 506.325 | 196.000 | 30.0 | -30.000 |
|  | 20:2 | 504.310 | 196.000 | 30.0 | -30.000 |
|  | 20:4 | 500.278 | 196.000 | 30.0 | -30.000 |
|  | 20:5 | 498.263 | 196.000 | 30.0 | -30.000 |
|  | 22:1 | 534.357 | 196.000 | 30.0 | -30.000 |
|  | 22:3 | 530.325 | 196.000 | 30.0 | -30.000 |
|  | 22:4 | 528.310 | 196.000 | 30.0 | -30.000 |
|  | 22:5 | 526.294 | 196.000 | 30.0 | -30.000 |
|  | 22:6 | 524.278 | 196.000 | 30.0 | -30.000 |
|  | 24:4 | 556.341 | 196.000 | 30.0 | -30.000 |
| O-LPE | 16:0 | 438.299 | 196.000 | 30.0 | -30.000 |
|  | 16:1 | 436.283 | 196.000 | 30.0 | -30.000 |
|  | 16:4 | 430.236 | 196.000 | 30.0 | -30.000 |
|  | 18:0 | 466.330 | 196.000 | 30.0 | -30.000 |
|  | 18:1 | 464.315 | 196.000 | 30.0 | -30.000 |
|  | 18:2 | 462.299 | 196.000 | 30.0 | -30.000 |
|  | 18:3 | 460.283 | 196.000 | 30.0 | -30.000 |
|  | 20:3 | 488.315 | 196.000 | 30.0 | -30.000 |
|  | 20:4 | 486.299 | 196.000 | 30.0 | -30.000 |
|  | 22:6 | 508.283 | 196.000 | 30.0 | -30.000 |
| LPS | 16:0 | 496.268 | 409.170 | 30.0 | -30.000 |
|  | 17:1 IS | 508.268 | 421.170 | 30.0 | -30.000 |
|  | 18:0 | 524.299 | 437.200 | 30.0 | -30.000 |
|  | 18:1 | 522.284 | 435.180 | 30.0 | -30.000 |
|  | 18:2 | 520.268 | 433.170 | 30.0 | -30.000 |
|  | 20:1 | 550.315 | 463.220 | 30.0 | -30.000 |
|  | 20:4 | 544.268 | 457.170 | 30.0 | -30.000 |
|  | 22:0 | 580.362 | 493.260 | 30.0 | -30.000 |
|  | 22:4 | 572.299 | 485.200 | 30.0 | -30.000 |
|  | 22:5 | 570.284 | 483.180 | 30.0 | -30.000 |
|  | 22:6 | 568.268 | 481.170 | 30.0 | -30.000 |
|  | 24:0 | 608.393 | 521.290 | 30.0 | -30.000 |
|  | 24:1 | 606.378 | 519.280 | 30.0 | -30.000 |
| LPG | 16:0 | 483.273 | 255.233 | 30.0 | -30.000 |
|  | 16:1 | 481.257 | 253.217 | 30.0 | -30.000 |
|  | 17:1 IS | 495.273 | 267.233 | 30.0 | -30.000 |
|  | 18:0 | 511.304 | 283.264 | 30.0 | -30.000 |
|  | 18:1 | 509.289 | 281.249 | 30.0 | -30.000 |
|  | 18:2 | 507.273 | 279.233 | 30.0 | -30.000 |
|  | 18:3 | 505.257 | 277.217 | 30.0 | -30.000 |
|  | 20:1 | 537.320 | 309.280 | 30.0 | -30.000 |
|  | 20:2 | 535.304 | 307.264 | 30.0 | -30.000 |
|  | 20:3 | 533.289 | 305.249 | 30.0 | -30.000 |
|  | 20:4 | 531.273 | 303.233 | 30.0 | -30.000 |
|  | 20:5 | 529.257 | 301.217 | 30.0 | -30.000 |
|  | 22:4 | 559.304 | 331.264 | 30.0 | -30.000 |
|  | 22:5 | 557.289 | 329.247 | 30.0 | -30.000 |
|  | 22:6 | 555.273 | 327.233 | 30.0 | -30.000 |
| LPI | 16:0 | 571.289 | 241.012 | 30.0 | -45.000 |
|  | 16:1 | 569.273 | 241.012 | 30.0 | -45.000 |
|  | 17:1 IS | 583.289 | 241.012 | 30.0 | -45.000 |
|  | 18:0 | 599.320 | 241.012 | 30.0 | -45.000 |
|  | 18:1 | 597.305 | 241.012 | 30.0 | -45.000 |
|  | 18:2 | 595.289 | 241.012 | 30.0 | -45.000 |
|  | 20:4 | 619.289 | 241.012 | 30.0 | -45.000 |
| SPBP | 14:0 | 324.195 | 78.900 | 30.0 | -35.000 |
|  | 16:0 | 352.226 | 78.900 | 30.0 | -35.000 |
|  | 16:1 | 350.210 | 78.900 | 30.0 | -35.000 |
|  | 16:2 | 348.195 | 78.900 | 30.0 | -35.000 |
|  | 17:1 IS | 364.226 | 78.900 | 30.0 | -35.000 |
|  | 18:0 | 380.257 | 78.900 | 30.0 | -35.000 |
|  | 18:1 | 378.241 | 78.900 | 30.0 | -35.000 |
|  | 18:2 | 376.226 | 78.900 | 30.0 | -35.000 |
|  | 20:0 | 408.288 | 78.900 | 30.0 | -35.000 |
|  | 20:1 | 406.273 | 78.900 | 30.0 | -35.000 |
|  | 20:2 | 404.257 | 78.900 | 30.0 | -35.000 |

## *S2.4 Ceramides and Sphingosine*


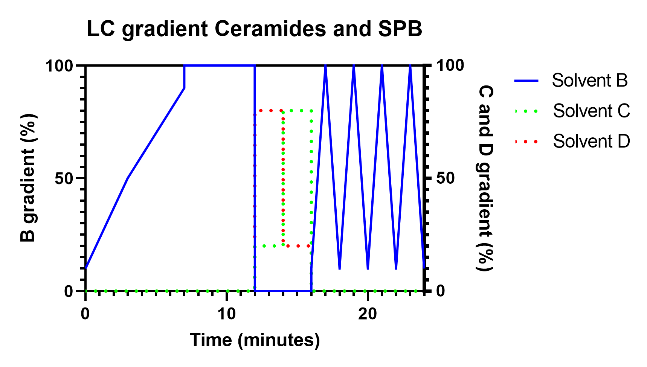
The solvent gradient was programmed as follows (**Figure S-6**): it started with 10% B, then increased in 3 minutes to 50%, and in 7 more minutes until 90%. At 10 minutes, it was directly increased to 100% B. At minute 12, pumps A and B were switched off and pumps C and D were switched on at a flow rate of 0.05 ml/min and 0.2 ml/min, respectively. They were maintained isocratic until minute 14, in which pump C was changed to a flow rate of 0.2 ml/min and pump D to 0.05 ml/min. At minute 16, pumps A and B were switched on, and C and D were switched off again. Gradient B was increased from 10% at minute 16 to 100% at minute 17. It was decreased to 10% in one minute and increased to 100% in the following minute for three times more, finishing at minute 24 at 10% of B. The oven temperature was set to 50 °C. The flow rate was set to 0.25 mL/min and it was directed to a QTRAP mass spectrometer. The mass spectrometer was operated in positive-ion mode and the ESI conditions were used as follows: curtain gas, 20.0; collision gas, medium; ion spray voltage, 4500 V; temperature, 400 ⁰C; ion source gas 1, 40.0; ion source gas 2, 30.0; declustering potential, 110; entrance potential, 10.0; and collision cell exit potential, 6.0. MS/MS analysis was carried out in MRM scanning mode, using the chosen transitions to fix the precursor ion and product ion *m/z* values at the first and third quadrupole, respectively (**Table S-4**). The total scan time was 1.68 seconds, dwell time was 10.0 milliseconds, and the collision energy was 30 V at the second quadrupole for all transitions. Data acquisition was carried out on Analyst (version 1.7.1). SCIEX OS (Version 2.1.6.59781) was used for analysing the data.

### **Figure S-6. LC method ceramides and SPB**

### **Table S-4. MRM transitions, dwell time and collision energies used for ceramides, dihydroceramides, and sphingosine LC-MS/MS analysis**

| **Lipid subclass** | **Molecular species** | **Q1 Mass (Da)** | **Q3 Mass (Da)** | **Dwell Time (msec)** | **CE (volts)** |
| --- | --- | --- | --- | --- | --- |
| Cer | d18:1/14:0 | 510.500 | 264.400 | 10.0 | 30.000 |
|  | d18:1/14:1 | 508.500 | 264.400 | 10.0 | 30.000 |
|  | d18:1/16:0 | 538.500 | 264.400 | 10.0 | 30.000 |
|  | d18:1/16:1 | 536.500 | 264.400 | 10.0 | 30.000 |
|  | d18:1/17:0 IS | 552.510 | 264.400 | 10.0 | 30.000 |
|  | d18:1/18:0 | 566.600 | 264.400 | 10.0 | 30.000 |
|  | d18:1/18:1 | 564.500 | 264.400 | 10.0 | 30.000 |
|  | d18:1/18:2 | 562.500 | 264.400 | 10.0 | 30.000 |
|  | d18:1/20:0 | 594.600 | 264.400 | 10.0 | 30.000 |
|  | d18:1/20:1 | 592.600 | 264.400 | 10.0 | 30.000 |
|  | d18:1/20:2 | 590.600 | 264.400 | 10.0 | 30.000 |
|  | d18:1/20:3 | 588.500 | 264.400 | 10.0 | 30.000 |
|  | d18:1/20:4 | 586.500 | 264.400 | 10.0 | 30.000 |
|  | d18:1/20:5 | 584.500 | 264.400 | 10.0 | 30.000 |
|  | d18:2/16:0 | 536.700 | 262.400 | 10.0 | 30.000 |
|  | d18:2/16:1 | 534.700 | 262.400 | 10.0 | 30.000 |
|  | d18:2/18:0 | 564.700 | 262.400 | 10.0 | 30.000 |
|  | d18:2/18:1 | 562.700 | 262.400 | 10.0 | 30.000 |
|  | d18:2/18:2 | 560.700 | 262.400 | 10.0 | 30.000 |
|  | d18:2/20:0 | 592.700 | 262.400 | 10.0 | 30.000 |
|  | d18:2/20:1 | 590.700 | 262.400 | 10.0 | 30.000 |
|  | d18:2/20:2 | 588.700 | 262.400 | 10.0 | 30.000 |
|  | d18:2/20:3 | 586.700 | 262.400 | 10.0 | 30.000 |
|  | d18:2/20:4 | 584.700 | 262.400 | 10.0 | 30.000 |
|  | d18:2/20:5 | 582.700 | 262.400 | 10.0 | 30.000 |
|  | d18:2/22:0 | 620.700 | 262.400 | 10.0 | 30.000 |
|  | d18:2/24:1 | 646.700 | 262.400 | 10.0 | 30.000 |
| dhCer | d18:0/14:0 | 508.500 | 266.400 | 10.0 | 30.000 |
|  | d18:0/16:0 | 540.500 | 266.400 | 10.0 | 30.000 |
|  | d18:0/16:1 | 538.500 | 266.400 | 10.0 | 30.000 |
|  | d18:0/18:0 | 568.600 | 266.400 | 10.0 | 30.000 |
|  | d18:0/18:1 | 566.600 | 266.400 | 10.0 | 30.000 |
|  | d18:0/18:2 | 564.600 | 266.400 | 10.0 | 30.000 |
|  | d18:0/20:0 | 596.600 | 266.400 | 10.0 | 30.000 |
|  | d18:0/20:1 | 594.600 | 266.400 | 10.0 | 30.000 |
|  | d18:0/20:2 | 592.600 | 266.400 | 10.0 | 30.000 |
|  | d18:0/20:3 | 590.600 | 266.400 | 10.0 | 30.000 |
|  | d18:0/20:4 | 588.600 | 266.400 | 10.0 | 30.000 |
|  | d18:0/20:5 | 586.600 | 266.400 | 10.0 | 30.000 |
|  | d18:0/22:0 | 624.600 | 266.400 | 10.0 | 30.000 |
|  | d18:0/24:0 | 652.700 | 266.400 | 10.0 | 30.000 |
| SPB and dhSPB | 17:1 IS | 286.310 | 238.300 | 10.0 | 30.000 |
|  | 18:0 | 302.300 | 284.300 | 10.0 | 30.000 |
|  | 18:1 | 300.300 | 282.300 | 10.0 | 30.000 |


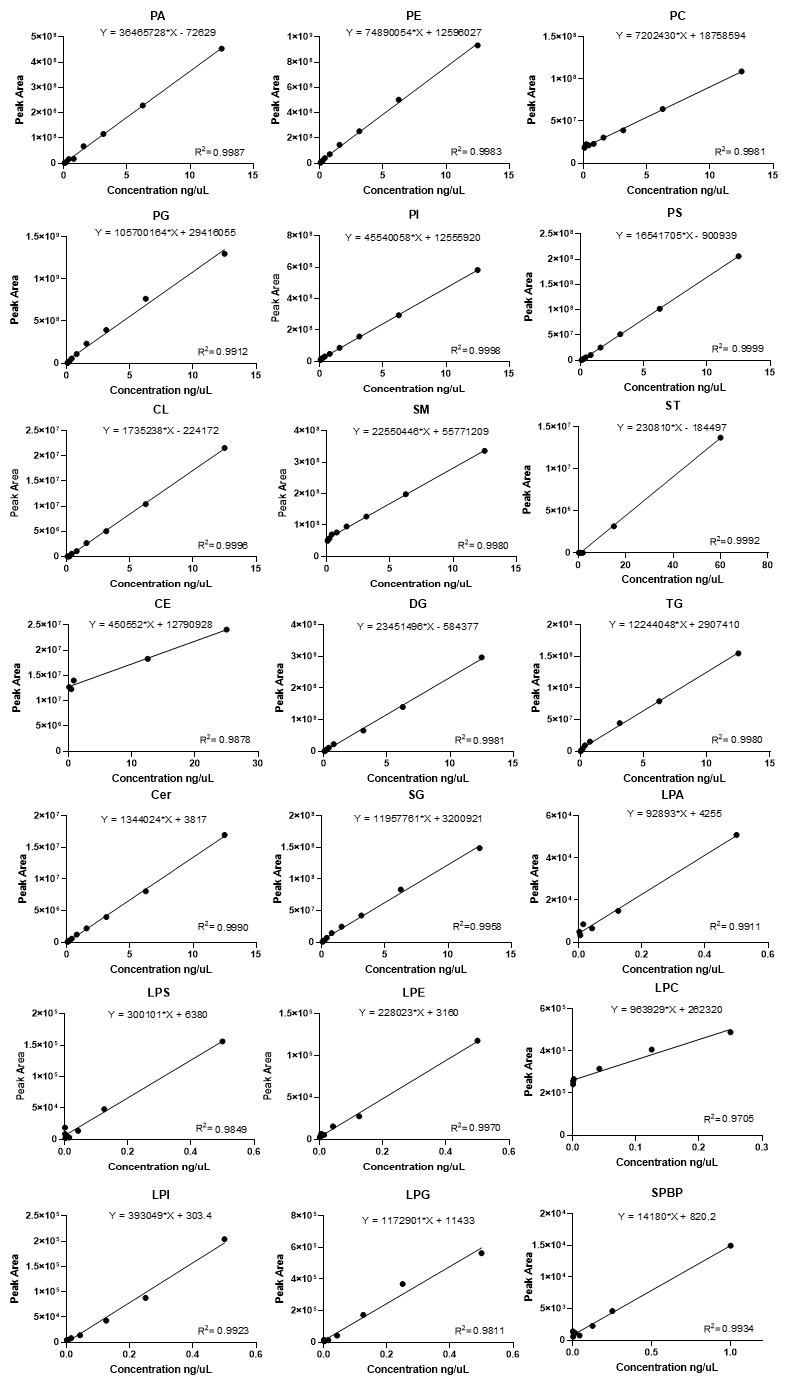


# Figure S-7. Calibration curves of selected internal standards spiked into SRM1950 plasma matrix for validation of linearity of HPLC/ESI-MS methods.

# Table S-5. Lipidome in SRM 1950.

| **Lipid molecular species** | **SRM1950 1** | **SRM1950 2** | **SRM1950 3** | **SRM1950 4** |
| --- | --- | --- | --- | --- |
| DG 32:0 | 0.031 | 0.026 | 0.025 | 0.019 |
| DG 32:1 | 0.046 | 0.043 | 0.045 | 0.058 |
| DG 32:2 | 0.000 | 0.000 | 0.040 | 0.000 |
| DG 34:0 | 0.013 | 0.000 | 0.000 | 0.000 |
| DG 34:1 | 0.352 | 0.365 | 0.374 | 0.370 |
| DG 34:2 | 0.292 | 0.297 | 0.344 | 0.300 |
| DG 36:1 | 0.055 | 0.043 | 0.048 | 0.044 |
| DG 36:2 | 0.501 | 0.498 | 0.555 | 0.543 |
| DG 36:3 | 0.530 | 0.585 | 0.599 | 0.580 |
| DG 36:4 | 0.000 | 0.000 | 0.000 | 0.000 |
| PC 30:0 | 0.290 | 0.256 | 0.267 | 0.266 |
| PC 30:1 | 0.046 | 0.042 | 0.043 | 0.039 |
| PC 32:0 | 1.258 | 1.147 | 1.266 | 1.355 |
| PC 32:1 | 1.919 | 1.756 | 0.000 | 2.093 |
| PC 32:2 | 0.428 | 0.368 | 0.402 | 0.444 |
| PC 34:1 | 0.000 | 14.382 | 20.978 | 21.862 |
| PC 34:2 | 40.895 | 36.441 | 41.335 | 44.662 |
| PC 34:3 | 0.000 | 0.027 | 0.000 | 1.489 |
| PC 34:4 | 0.075 | 0.064 | 0.083 | 0.063 |
| PC 36:1 | 2.838 | 2.848 | 3.123 | 0.000 |
| PC 36:2 | 19.623 | 18.708 | 21.511 | 21.328 |
| PC 36:3 | 13.872 | 12.178 | 13.986 | 12.810 |
| PC 36:4 | 17.610 | 15.796 | 18.412 | 19.316 |
| PC 36:5 | 1.114 | 1.050 | 1.151 | 1.268 |
| PC 36:6 | 0.023 | 0.023 | 0.026 | 0.023 |
| PC 38:1 | 0.027 | 0.028 | 0.030 | 0.034 |
| PC 38:3 | 3.371 | 3.477 | 4.037 | 3.984 |
| PC 38:4 | 10.422 | 10.766 | 11.508 | 11.804 |
| PC 38:5 | 4.751 | 4.359 | 4.993 | 4.923 |
| PC 38:6 | 4.413 | 4.033 | 4.594 | 4.695 |
| PC 38:7 | 0.074 | 0.062 | 0.075 | 0.071 |
| PC 40:1 | 0.005 | 0.005 | 0.005 | 0.000 |
| PC 40:2 | 0.013 | 0.010 | 0.014 | 0.012 |
| PC 40:4 | 0.457 | 0.410 | 0.461 | 0.472 |
| PC 40:5 | 0.906 | 0.944 | 1.151 | 1.078 |
| PC 40:6 | 1.617 | 1.527 | 1.664 | 1.610 |
| PC 40:7 | 0.313 | 0.307 | 0.370 | 0.347 |
| PE 36:2 | 0.294 | 0.398 | 0.459 | 0.489 |
| PE 36:3 | 0.079 | 0.020 | 0.022 | 0.024 |
| PE 36:4 | 0.000 | 0.201 | 0.218 | 0.277 |
| PE 36:5 | 0.000 | 0.000 | 0.000 | 0.021 |
| PE 38:3 | 0.049 | 0.024 | 0.000 | 0.635 |
| PE 38:4 | 0.333 | 0.633 | 0.643 | 0.073 |
| PE 38:5 | 0.635 | 0.170 | 0.071 | 0.216 |
| PE 38:6 | 0.106 | 0.182 | 0.206 | 0.011 |
| PE 40:4 | 0.000 | 0.010 | 0.014 | 0.036 |
| PE 40:5 | 0.339 | 0.141 | 0.038 | 0.015 |
| PE 40:6 | 0.287 | 0.100 | 0.015 | 0.022 |
| PE 40:7 | 0.183 | 0.061 | 0.027 | 0.000 |
| PI 32:0 | 0.041 | 0.032 | 0.041 | 0.038 |
| PI 32:1 | 0.142 | 0.126 | 0.153 | 0.132 |
| PI 34:1 | 0.959 | 0.860 | 1.052 | 0.892 |
| PI 34:2 | 0.931 | 0.842 | 1.033 | 0.896 |
| PI 34:3 | 0.015 | 0.014 | 0.016 | 0.012 |
| PI 36:1 | 0.000 | 0.000 | 0.000 | 0.000 |
| PI 36:2 | 3.223 | 2.922 | 3.274 | 3.156 |
| PI 36:3 | 0.665 | 0.604 | 0.708 | 0.610 |
| PI 36:4 | 0.865 | 0.826 | 0.994 | 0.802 |
| PI 36:5 | 0.006 | 0.004 | 0.005 | 0.006 |
| PI 38:4 | 8.511 | 7.774 | 9.208 | 8.208 |
| PI 38:5 | 0.722 | 0.644 | 0.755 | 0.675 |
| PI 38:6 | 0.074 | 0.064 | 0.082 | 0.068 |
| PI 40:4 | 0.090 | 0.074 | 0.095 | 0.078 |
| PI 40:5 | 0.230 | 0.200 | 0.237 | 0.195 |
| PI 40:6 | 0.258 | 0.221 | 0.268 | 0.222 |
| PI 40:7 | 0.010 | 0.009 | 0.012 | 0.008 |
| PG 34:1 | 0.006 | 0.004 | 0.005 | 0.004 |
| PG 34:2 | 0.001 | 0.001 | 0.001 | 0.001 |
| PG 36:1 | 0.006 | 0.004 | 0.005 | 0.004 |
| PG 36:2 | 0.005 | 0.004 | 0.005 | 0.004 |
| PG 36:3 | 0.001 | 0.000 | 0.001 | 0.000 |
| PG 36:4 | 0.000 | 0.000 | 0.000 | 0.000 |
| PG 38:2 | 0.000 | 0.000 | 0.000 | 0.000 |
| SM (d18:1/16:0) | 13.473 | 12.365 | 14.338 | 15.293 |
| SM (d18:1/16:1) | 1.926 | 1.674 | 2.125 | 2.264 |
| SM (d18:1/18:0) | 2.662 | 2.624 | 2.367 | 3.396 |
| SM (d18:1/18:1) | 1.255 | 1.301 | 1.566 | 1.711 |
| SM (d18:1/18:2) | 0.130 | 0.130 | 0.127 | 0.163 |
| SM (d18:1/18:3) | 0.006 | 0.006 | 0.006 | 0.005 |
| SM (d18:1/20:0) | 1.560 | 1.388 | 1.601 | 1.944 |
| SM (d18:1/20:1) | 0.667 | 0.599 | 0.691 | 0.856 |
| SM (d18:1/20:2) | 0.043 | 0.047 | 0.053 | 0.055 |
| SM (d18:1/20:3) | 0.005 | 0.005 | 0.005 | 0.005 |
| SM (d18:1/22:0) | 3.033 | 2.527 | 3.144 | 3.629 |
| SM (d18:1/22:1) | 2.107 | 1.890 | 2.286 | 2.705 |
| SM (d18:1/24:0) | 1.801 | 1.512 | 1.981 | 2.091 |
| SM (d18:1/24:1) | 4.551 | 4.423 | 4.906 | 5.763 |
| SM (d18:1/24:5) | 0.009 | 0.009 | 0.008 | 0.011 |
| TG 36:0 | 0.006 | 0.002 | 0.004 | 0.000 |
| TG 36:1 | 0.010 | 0.007 | 0.009 | 0.009 |
| TG 38:0 | 0.018 | 0.010 | 0.015 | 0.015 |
| TG 38:1 | 0.036 | 0.028 | 0.033 | 0.049 |
| TG 38:2 | 0.010 | 0.006 | 0.009 | 0.006 |
| TG 40:0 | 0.035 | 0.028 | 0.032 | 0.055 |
| TG 40:1 | 0.036 | 0.027 | 0.034 | 0.052 |
| TG 40:2 | 0.019 | 0.013 | 0.017 | 0.019 |
| TG 40:3 | 0.003 | 0.002 | 0.003 | 0.000 |
| TG 42:0 | 0.077 | 0.062 | 0.069 | 0.110 |
| TG 42:1 | 0.075 | 0.062 | 0.070 | 0.118 |
| TG 42:2 | 0.039 | 0.027 | 0.029 | 0.058 |
| TG 42:3 | 0.006 | 0.004 | 0.004 | 0.009 |
| TG 44:0 | 0.239 | 0.196 | 0.224 | 0.319 |
| TG 44:1 | 0.216 | 0.177 | 0.195 | 0.306 |
| TG 44:2 | 0.107 | 0.093 | 0.101 | 0.165 |
| TG 44:3 | 0.029 | 0.025 | 0.029 | 0.039 |
| TG 46:0 | 0.709 | 0.684 | 0.724 | 1.152 |
| TG 46:1 | 0.936 | 0.816 | 0.945 | 1.255 |
| TG 46:2 | 0.423 | 0.358 | 0.401 | 0.597 |
| TG 46:3 | 0.120 | 0.104 | 0.113 | 0.165 |
| TG 48:0 | 1.208 | 1.475 | 1.422 | 2.763 |
| TG 48:1 | 3.634 | 3.751 | 4.014 | 6.121 |
| TG 48:2 | 2.812 | 2.589 | 3.024 | 3.937 |
| TG 48:3 | 0.791 | 0.714 | 0.799 | 1.053 |
| TG 48:4 | 0.181 | 0.157 | 0.178 | 0.234 |
| TG 48:5 | 0.033 | 0.026 | 0.029 | 0.035 |
| TG 50:0 | 0.672 | 0.830 | 0.823 | 1.960 |
| TG 50:1 | 7.826 | 9.535 | 9.198 | 17.669 |
| TG 50:2 | 11.355 | 12.308 | 12.994 | 19.745 |
| TG 50:3 | 6.285 | 5.803 | 6.867 | 8.786 |
| TG 50:4 | 1.449 | 1.348 | 1.486 | 1.932 |
| TG 50:5 | 0.257 | 0.233 | 0.263 | 0.323 |
| TG 50:6 | 0.000 | 0.000 | 0.000 | 0.000 |
| TG 50:7 | 0.000 | 0.000 | 0.000 | 0.000 |
| TG 52:0 | 0.273 | 0.236 | 0.286 | 0.368 |
| TG 52:1 | 2.320 | 2.906 | 2.853 | 6.473 |
| TG 52:2 | 19.937 | 24.031 | 23.192 | 42.488 |
| TG 52:3 | 24.101 | 26.579 | 28.119 | 47.533 |
| TG 52:4 | 14.146 | 13.476 | 15.561 | 19.646 |
| TG 52:5 | 2.855 | 2.751 | 2.977 | 3.917 |
| TG 52:6 | 0.442 | 0.402 | 0.445 | 0.592 |
| TG 52:7 | 0.063 | 0.051 | 0.048 | 0.064 |
| TG 52:8 | 0.007 | 0.005 | 0.006 | 0.006 |
| TG 54:0 | 0.044 | 0.029 | 0.040 | 0.039 |
| TG 54:1 | 0.492 | 0.440 | 0.514 | 0.725 |
| TG 54:2 | 2.045 | 2.440 | 2.474 | 5.120 |
| TG 54:3 | 7.712 | 8.981 | 8.777 | 14.720 |
| TG 54:4 | 8.363 | 9.012 | 9.512 | 16.354 |
| TG 54:5 | 1.535 | 1.595 | 1.657 | 2.542 |
| TG 54:6 | 1.535 | 1.452 | 1.664 | 2.079 |
| TG 54:7 | 0.429 | 0.298 | 0.417 | 0.512 |
| TG 54:8 | 0.079 | 0.085 | 0.097 | 0.124 |
| TG 54:9 | 0.010 | 0.008 | 0.009 | 0.008 |
| TG 56:0 | 0.020 | 0.005 | 0.009 | 0.011 |
| TG 56:1 | 0.068 | 0.047 | 0.071 | 0.083 |
| TG 56:10 | 0.016 | 0.011 | 0.014 | 0.014 |
| TG 56:2 | 0.169 | 0.141 | 0.169 | 0.223 |
| TG 56:3 | 0.205 | 0.230 | 0.234 | 0.445 |
| TG 56:4 | 0.452 | 0.474 | 0.471 | 0.677 |
| TG 56:5 | 0.969 | 1.024 | 1.076 | 1.221 |
| TG 56:6 | 2.124 | 2.134 | 2.304 | 3.065 |
| TG 56:7 | 1.030 | 0.892 | 1.050 | 1.160 |
| TG 56:8 | 0.396 | 0.388 | 0.417 | 0.506 |
| TG 56:9 | 0.132 | 0.109 | 0.124 | 0.141 |
| TG 58:0 | 0.008 | 0.000 | 0.002 | 0.000 |
| TG 58:1 | 0.048 | 0.028 | 0.036 | 0.051 |
| TG 58:2 | 0.082 | 0.057 | 0.084 | 0.105 |
| TG 58:3 | 0.073 | 0.063 | 0.074 | 0.095 |
| TG 58:4 | 0.037 | 0.035 | 0.039 | 0.064 |
| TG 58:5 | 0.061 | 0.059 | 0.067 | 0.081 |
| TG 58:6 | 0.105 | 0.123 | 0.122 | 0.219 |
| TG 58:7 | 0.253 | 0.275 | 0.309 | 0.401 |
| TG 58:8 | 0.337 | 0.296 | 0.359 | 0.384 |
| TG 58:9 | 0.228 | 0.207 | 0.239 | 0.237 |
| CE 16:0 | 2.565 | 2.562 | 2.687 | 5.078 |
| CE 16:1 | 1.221 | 1.190 | 1.159 | 2.601 |
| CE 18:0 | 1.187 | 0.885 | 1.069 | 1.019 |
| CE 18:1 | 11.736 | 12.081 | 12.300 | 25.684 |
| CE 18:2 | 25.586 | 27.887 | 24.972 | 67.971 |
| CE 18:3 | 3.145 | 2.607 | 2.947 | 4.675 |
| CE 20:2 | 0.176 | 0.151 | 0.174 | 0.173 |
| CE 20:3 | 1.119 | 1.072 | 1.129 | 2.085 |
| CE 20:4 | 13.646 | 11.627 | 12.586 | 23.991 |
| CE 20:5 | 1.569 | 1.145 | 1.348 | 1.123 |
| CE 22:4 | 0.000 | 0.000 | 0.000 | 0.000 |
| CE 22:5 | 0.314 | 0.193 | 0.171 | 0.287 |
| CE 22:6 | 2.354 | 1.780 | 2.577 | 2.451 |
| ST 27:0 | 39.381 | 26.883 | 37.291 | 27.735 |
| O-PC 32:0 | 0.118 | 0.105 | 0.118 | 0.117 |
| O-PC 32:1/P-PC 32:0 | 0.137 | 0.119 | 0.142 | 0.140 |
| O-PC 32:2/P-PC 32:1 | 0.017 | 0.013 | 0.015 | 0.016 |
| O-PC 34:1/P-PC 34:0 | 0.229 | 0.220 | 0.294 | 0.250 |
| O-PC 34:2/P-PC 34:1 | 0.526 | 0.485 | 0.570 | 0.537 |
| O-PC 34:3/P-PC 34:2 | 0.000 | 0.468 | 0.564 | 0.592 |
| O-PC 36:2/P-PC 36:1 | 0.221 | 0.178 | 0.206 | 0.206 |
| O-PC 36:4/P-PC 36:3 | 1.351 | 1.288 | 1.275 | 1.201 |
| O-PC 36:5/P-PC 36:4 | 0.999 | 0.794 | 0.938 | 0.999 |
| O-PC 36:6/P-PC 36:5 | 0.000 | 0.012 | 0.017 | 0.012 |
| O-PC 38:1/P-PC 38:0 | 0.014 | 0.012 | 0.015 | 0.014 |
| O-PC 38:2/P-PC 38:1 | 0.032 | 0.000 | 0.034 | 0.040 |
| O-PC 38:4/P-PC 38:3 | 1.315 | 1.059 | 1.275 | 1.310 |
| O-PC 38:5/P-PC 38:4 | 1.941 | 1.668 | 2.160 | 2.066 |
| O-PC 38:6/P-PC 38:5 | 0.367 | 0.538 | 0.643 | 0.678 |
| O-PC 38:7/P-PC 38:6 | 0.056 | 0.059 | 0.068 | 0.068 |
| O-PC 40:1/P-PC 40:0 | 0.014 | 0.011 | 0.016 | 0.000 |
| O-PC 40:2/P-PC 40:1 | 0.033 | 0.025 | 0.035 | 0.028 |
| O-PC 40:4/P-PC 40:3 | 0.287 | 0.235 | 0.301 | 0.283 |
| O-PC 40:5/P-PC 40:4 | 0.475 | 0.397 | 0.520 | 0.494 |
| O-PC 40:6/P-PC 40:5 | 0.356 | 0.291 | 0.387 | 0.375 |
| O-PC 40:7/P-PC 40:6 | 0.199 | 0.176 | 0.209 | 0.217 |
| O-PC 40:8/P-PC 40:7 | 0.024 | 0.023 | 0.029 | 0.026 |
| O-PE 34:1/P-PE 34:0 | 0.000 | 0.007 | 0.012 | 0.000 |
| O-PE 34:2/P-PE 34:1 | 0.037 | 0.042 | 0.053 | 0.000 |
| O-PE 34:3/P-PE 34:2 | 0.082 | 0.115 | 0.130 | 0.000 |
| O-PE 34:4/P-PE 34:3 | 0.000 | 0.000 | 0.000 | 0.000 |
| O-PE 36:2/P-PE 36:1 | 0.046 | 0.000 | 0.000 | 0.000 |
| O-PE 36:3/P-PE 36:2 | 0.230 | 0.250 | 0.286 | 0.000 |
| O-PE 36:4/P-PE 36:3 | 0.104 | 0.190 | 0.197 | 0.000 |
| O-PE 36:5/P-PE 36:4 | 0.306 | 0.469 | 0.479 | 0.000 |
| O-PE 36:6/P-PE 36:5 | 0.000 | 0.019 | 0.017 | 0.000 |
| O-PE 38:2/P-PE 38:1 | 0.000 | 0.000 | 0.000 | 0.000 |
| O-PE 38:4/P-PE 38:3 | 0.000 | 0.000 | 0.000 | 0.000 |
| O-PE 38:5/P-PE 38:4 | 0.679 | 1.004 | 1.088 | 1.068 |
| O-PE 38:6/P-PE 38:5 | 0.348 | 0.682 | 0.687 | 0.676 |
| O-PE 38:7/P-PE 38:6 | 0.138 | 0.235 | 0.245 | 0.000 |
| O-PE 40:3/P-PE 40:2 | 0.000 | 0.000 | 0.000 | 0.000 |
| O-PE 40:5/P-PE 40:4 | 0.000 | 0.077 | 0.084 | 0.000 |
| O-PE 40:6/P-PE 40:5 | 0.061 | 0.140 | 0.146 | 0.000 |
| O-PE 40:7/P-PE 40:6 | 0.132 | 0.253 | 0.274 | 0.000 |
| O-PE 40:8/P-PE 40:7 | 0.000 | 0.106 | 0.117 | 0.000 |
| O-PE 40:9/P-PE 40:8 | 0.000 | 0.000 | 0.000 | 0.000 |
| O-DG 30:2 | 0.101 | 0.122 | 0.241 | 0.357 |
| O-TG 50:0 | 0.004 | 0.003 | 0.004 | 0.000 |
| O-TG 50:1 | 0.026 | 0.021 | 0.027 | 0.026 |
| O-TG 50:2 | 0.008 | 0.007 | 0.008 | 0.007 |
| O-TG 52:1 | 0.013 | 0.008 | 0.011 | 0.012 |
| O-TG 52:2 | 0.029 | 0.023 | 0.030 | 0.028 |
| O-TG 52:3 | 0.010 | 0.009 | 0.011 | 0.010 |
| O-TG 54:1 | 0.003 | 0.000 | 0.000 | 0.000 |
| O-TG 54:2 | 0.006 | 0.004 | 0.006 | 0.005 |
| O-TG 54:3 | 0.006 | 0.004 | 0.006 | 0.006 |
| O-TG 56:2 | 0.004 | 0.003 | 0.004 | 0.000 |
| LPC 14:0 | 0.025 | 0.024 | 0.017 | 0.019 |
| LPC 16:0 | 2.898 | 2.840 | 2.055 | 2.212 |
| LPC 16:1 | 0.197 | 0.191 | 0.135 | 0.169 |
| LPC 18:0 | 1.938 | 1.761 | 1.250 | 1.735 |
| LPC 18:1 | 1.196 | 1.097 | 0.755 | 1.012 |
| LPC 18:2 | 1.293 | 1.175 | 0.867 | 0.923 |
| LPC 18:3 | 0.032 | 0.029 | 0.022 | 0.022 |
| LPC 20:0 | 0.012 | 0.012 | 0.009 | 0.013 |
| LPC 20:1 | 0.025 | 0.025 | 0.016 | 0.022 |
| LPC 20:2 | 0.019 | 0.018 | 0.013 | 0.014 |
| LPC 20:3 | 0.143 | 0.141 | 0.098 | 0.134 |
| LPC 20:4 | 0.490 | 0.428 | 0.331 | 0.334 |
| LPC 20:5 | 0.019 | 0.021 | 0.013 | 0.021 |
| LPC 22:0 | 0.004 | 0.004 | 0.003 | 0.003 |
| LPC 22:1 | 0.003 | 0.003 | 0.002 | 0.002 |
| LPC 22:2 | 0.001 | 0.001 | 0.001 | 0.001 |
| LPC 22:3 | 0.001 | 0.001 | 0.001 | 0.001 |
| LPC 22:4 | 0.019 | 0.020 | 0.013 | 0.015 |
| LPC 22:5 | 0.044 | 0.042 | 0.028 | 0.038 |
| LPC 22:6 | 0.068 | 0.061 | 0.045 | 0.041 |
| LPC 24:0 | 0.005 | 0.005 | 0.004 | 0.005 |
| LPC 24:1 | 0.003 | 0.003 | 0.002 | 0.003 |
| LPC 26:0 | 0.002 | 0.001 | 0.001 | 0.001 |
| LPE 16:0 | 0.121 | 0.127 | 0.067 | 0.104 |
| LPE 16:1 | 0.005 | 0.004 | 0.003 | 0.005 |
| LPE 18:0 | 0.152 | 0.124 | 0.106 | 0.188 |
| LPE 18:1 | 0.092 | 0.085 | 0.063 | 0.095 |
| LPE 18:2 | 0.270 | 0.239 | 0.207 | 0.331 |
| LPE 20:1 | 0.003 | 0.003 | 0.002 | 0.003 |
| LPE 20:2 | 0.002 | 0.001 | 0.001 | 0.002 |
| LPE 20:4 | 0.092 | 0.076 | 0.061 | 0.099 |
| LPE 20:5 | 0.010 | 0.008 | 0.008 | 0.013 |
| LPE 22:5 | 0.010 | 0.010 | 0.007 | 0.008 |
| LPE 22:6 | 0.038 | 0.038 | 0.026 | 0.054 |
| LPS 18:0 | 0.002 | 0.001 | 0.001 | 0.001 |
| LPS 18:1 | 0.001 | 0.001 | 0.001 | 0.001 |
| LPS 18:2 | 0.000 | 0.001 | 0.000 | 0.000 |
| LPS 20:4 | 0.002 | 0.001 | 0.001 | 0.000 |
| LPS 22:6 | 0.001 | 0.001 | 0.001 | 0.000 |
| LPA 16:0 | 0.189 | 0.177 | 0.169 | 0.148 |
| LPA 16:1 | 0.007 | 0.007 | 0.007 | 0.008 |
| LPA 18:0 | 0.053 | 0.056 | 0.042 | 0.045 |
| LPA 18:1 | 0.025 | 0.023 | 0.016 | 0.023 |
| LPA 18:2 | 0.114 | 0.109 | 0.074 | 0.109 |
| LPA 18:3 | 0.003 | 0.002 | 0.002 | 0.003 |
| LPA 20:3 | 0.007 | 0.007 | 0.005 | 0.007 |
| LPA 20:4 | 0.041 | 0.038 | 0.030 | 0.037 |
| LPA 20:5 | 0.002 | 0.003 | 0.002 | 0.003 |
| LPA 22:5 | 0.002 | 0.003 | 0.002 | 0.002 |
| LPA 22:6 | 0.038 | 0.033 | 0.036 | 0.028 |
| LPI 16:0 | 0.011 | 0.011 | 0.007 | 0.009 |
| LPI 16:1 | 0.010 | 0.010 | 0.006 | 0.006 |
| LPI 18:0 | 0.046 | 0.043 | 0.033 | 0.032 |
| LPI 18:1 | 0.036 | 0.034 | 0.028 | 0.027 |
| LPI 18:2 | 0.046 | 0.050 | 0.033 | 0.029 |
| LPI 20:4 | 0.057 | 0.057 | 0.038 | 0.046 |
| LPG 16:0 | 0.065 | 0.057 | 0.041 | 0.054 |
| LPG 16:1 | 0.959 | 0.817 | 0.642 | 0.784 |
| LPG 18:0 | 0.026 | 0.021 | 0.018 | 0.019 |
| LPG 18:1 | 0.336 | 0.279 | 0.215 | 0.239 |
| LPG 18:2 | 0.252 | 0.204 | 0.174 | 0.194 |
| LPG 18:3 | 0.322 | 0.271 | 0.217 | 0.307 |
| LPG 20:1 | 0.001 | 0.001 | 0.001 | 0.001 |
| LPG 20:2 | 0.002 | 0.002 | 0.001 | 0.002 |
| LPG 20:3 | 0.004 | 0.003 | 0.003 | 0.004 |
| LPG 20:4 | 0.019 | 0.015 | 0.011 | 0.014 |
| LPG 20:5 | 0.034 | 0.028 | 0.022 | 0.033 |
| LPG 22:5 | 0.001 | 0.001 | 0.001 | 0.001 |
| LPG 22:6 | 0.003 | 0.003 | 0.002 | 0.002 |
| O-LPC 16:0 | 0.026 | 0.025 | 0.017 | 0.029 |
| O-LPC 18:0 | 0.115 | 0.111 | 0.077 | 0.085 |
| O-LPC 20:0 | 0.037 | 0.035 | 0.024 | 0.035 |
| O-LPE 16:0 | 0.004 | 0.002 | 0.002 | 0.003 |
| O-LPE 16:1 | 0.060 | 0.056 | 0.045 | 0.078 |
| O-LPE 18:0 | 0.002 | 0.003 | 0.001 | 0.002 |
| O-LPE 18:1 | 0.034 | 0.030 | 0.021 | 0.023 |
| O-LPE 18:2 | 0.028 | 0.026 | 0.021 | 0.030 |
| O-LPA 18:0 | 0.046 | 0.039 | 0.027 | 0.037 |
| P-LPA 16:0 | 0.003 | 0.002 | 0.002 | 0.002 |
| P-LPA 16:1 | 0.006 | 0.006 | 0.004 | 0.004 |
| P-LPA 18:0 | 0.034 | 0.032 | 0.024 | 0.028 |
| P-LPC 16:0 | 0.050 | 0.049 | 0.032 | 0.048 |
| P-LPC 16:1 | 0.001 | 0.000 | 0.000 | 0.000 |
| P-LPC 18:0 | 0.018 | 0.016 | 0.011 | 0.012 |
| P-LPC 18:1 | 0.001 | 0.001 | 0.001 | 0.001 |
| P-LPC 20:0 | 0.006 | 0.006 | 0.004 | 0.005 |
| P-LPC 20:1 | 0.001 | 0.001 | 0.001 | 0.001 |
| SPBP 18:0 | 0.041 | 0.033 | 0.032 | 0.022 |
| SPBP 18:1 | 0.124 | 0.098 | 0.111 | 0.071 |
| SPBP 18:2 | 0.063 | 0.061 | 0.063 | 0.036 |
| dhCer (d18:0/16:0) | 0.013 | 0.012 | 0.012 | 0.005 |
| dhCer (d18:0/18:0) | 0.002 | 0.002 | 0.002 | 0.037 |
| dhCer (d18:0/20:0) | 0.009 | 0.006 | 0.007 | 0.094 |
| dhCer (d18:0/22:0) | 0.030 | 0.023 | 0.027 | 0.229 |
| dhCer (d18:0/24:0) | 0.028 | 0.031 | 0.025 | 0.317 |
| dhSM (d18:0/16:0) | 0.452 | 0.401 | 0.451 | 0.550 |
| dhSM (d18:0/18:0) | 0.105 | 0.085 | 0.101 | 0.110 |
| dhSM (d18:0/20:0) | 0.018 | 0.016 | 0.018 | 0.022 |
| Cer (d18:1/14:0) | 0.003 | 0.002 | 0.003 | 0.040 |
| Cer (d18:1/16:0) | 0.068 | 0.056 | 0.061 | 0.455 |
| Cer (d18:1/18:0) | 0.013 | 0.010 | 0.012 | 0.059 |
| Cer (d18:1/18:1) | 0.004 | 0.003 | 0.004 | 0.020 |
| Cer (d18:1/20:1) | 0.003 | 0.004 | 0.004 | 0.004 |
| Cer (d18:1/22:0) | 0.066 | 0.061 | 0.070 | 0.695 |
| Cer (d18:1/22:2) | 0.004 | 0.004 | 0.005 | 0.067 |
| Cer (d18:1/24:0) | 0.118 | 0.129 | 0.107 | 1.386 |
| Cer (d18:1/24:1) | 0.077 | 0.073 | 0.070 | 0.752 |
| Cer (d18:2/16:0) | 0.025 | 0.010 | 0.012 | 0.131 |
| Cer (d18:2/22:0) | 0.012 | 0.012 | 0.013 | 0.141 |
| Cer (d18:2/24:1) | 0.016 | 0.015 | 0.017 | 0.163 |


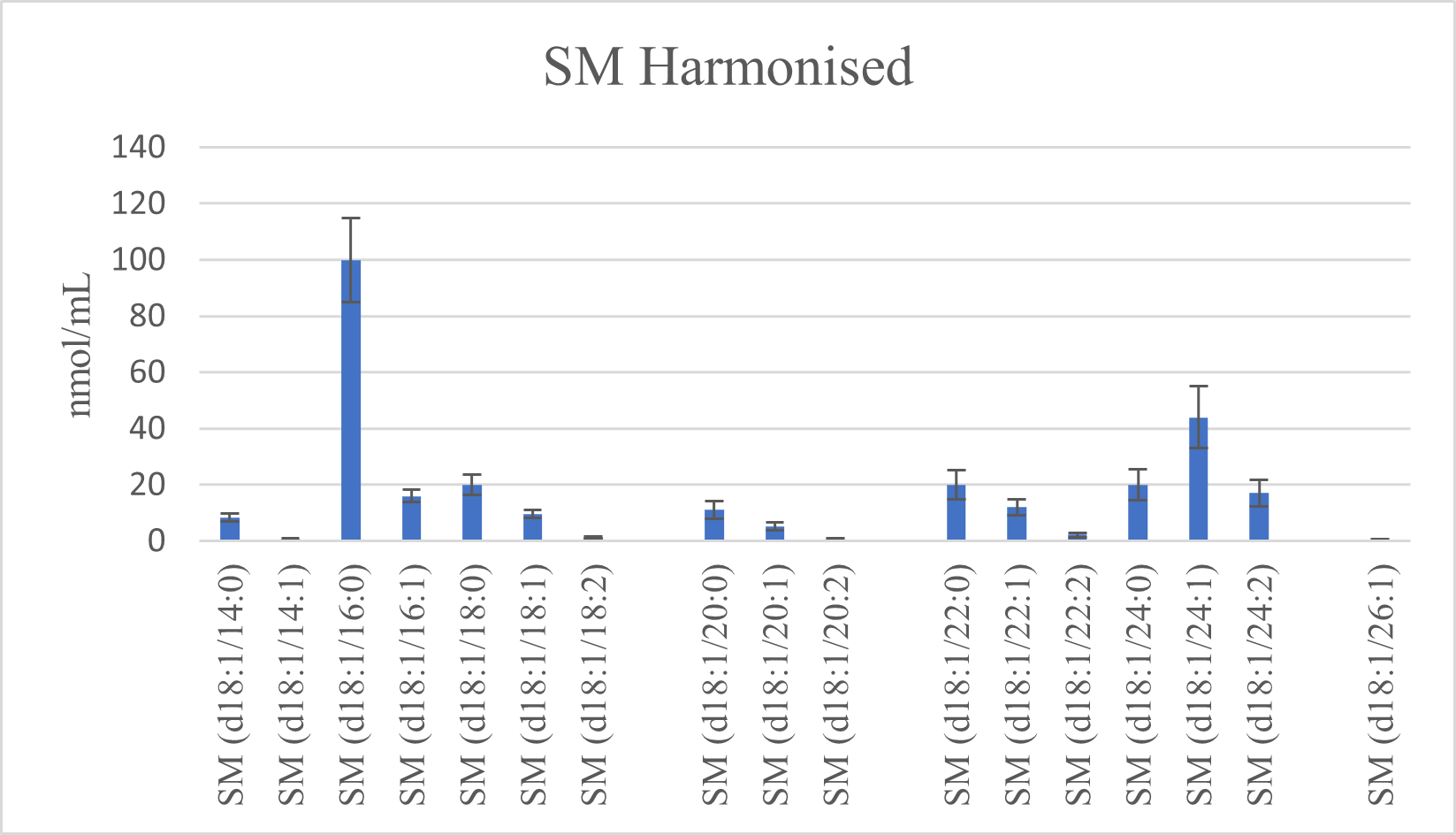


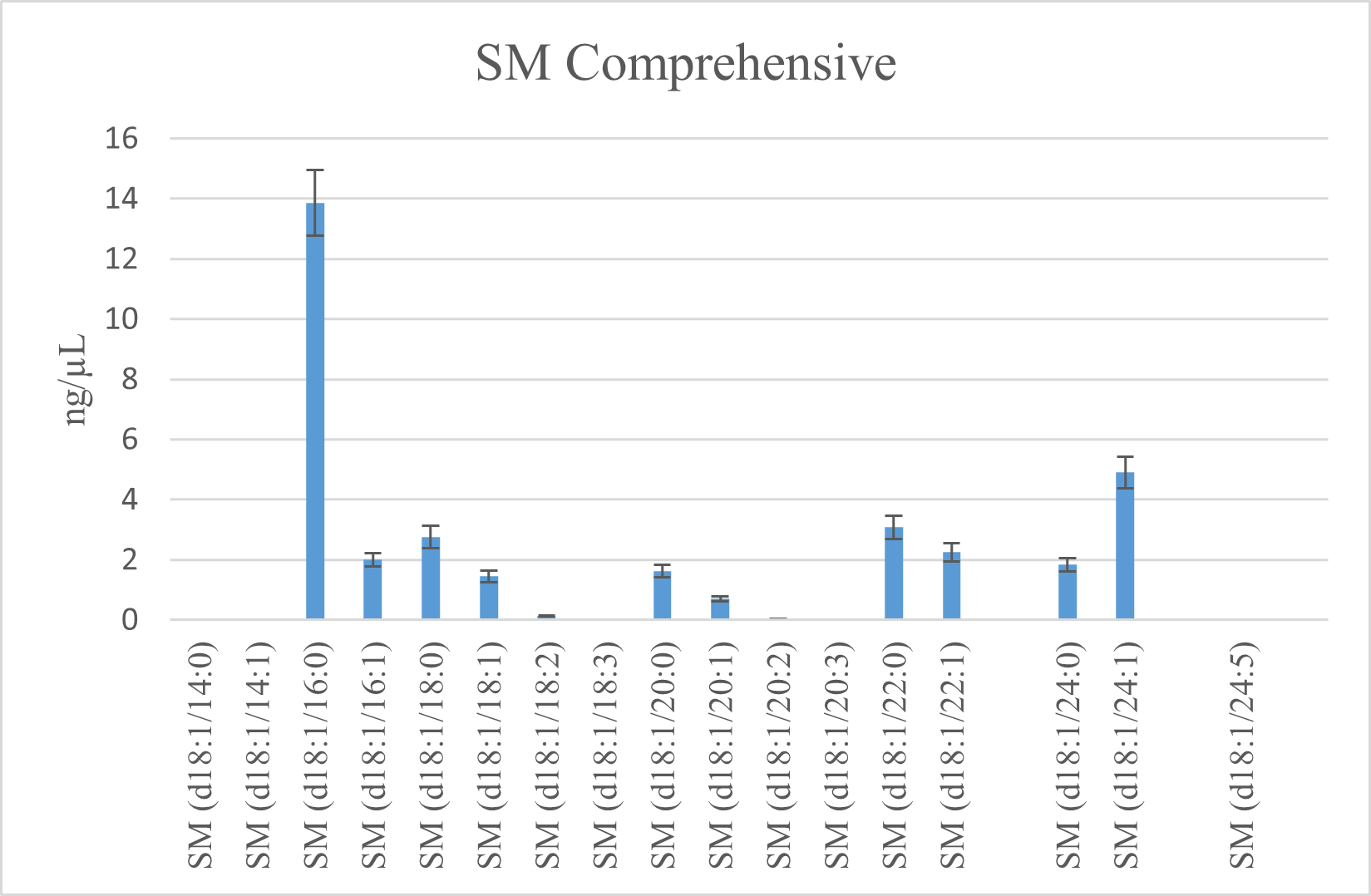


# Figure S-8. Sphingomyelin (SM) profile obtained in human plasma SRM 1950: SM comprehensive are the results produced with the GP method described here. SM harmonised are the results obtained by Bowden *et al.*


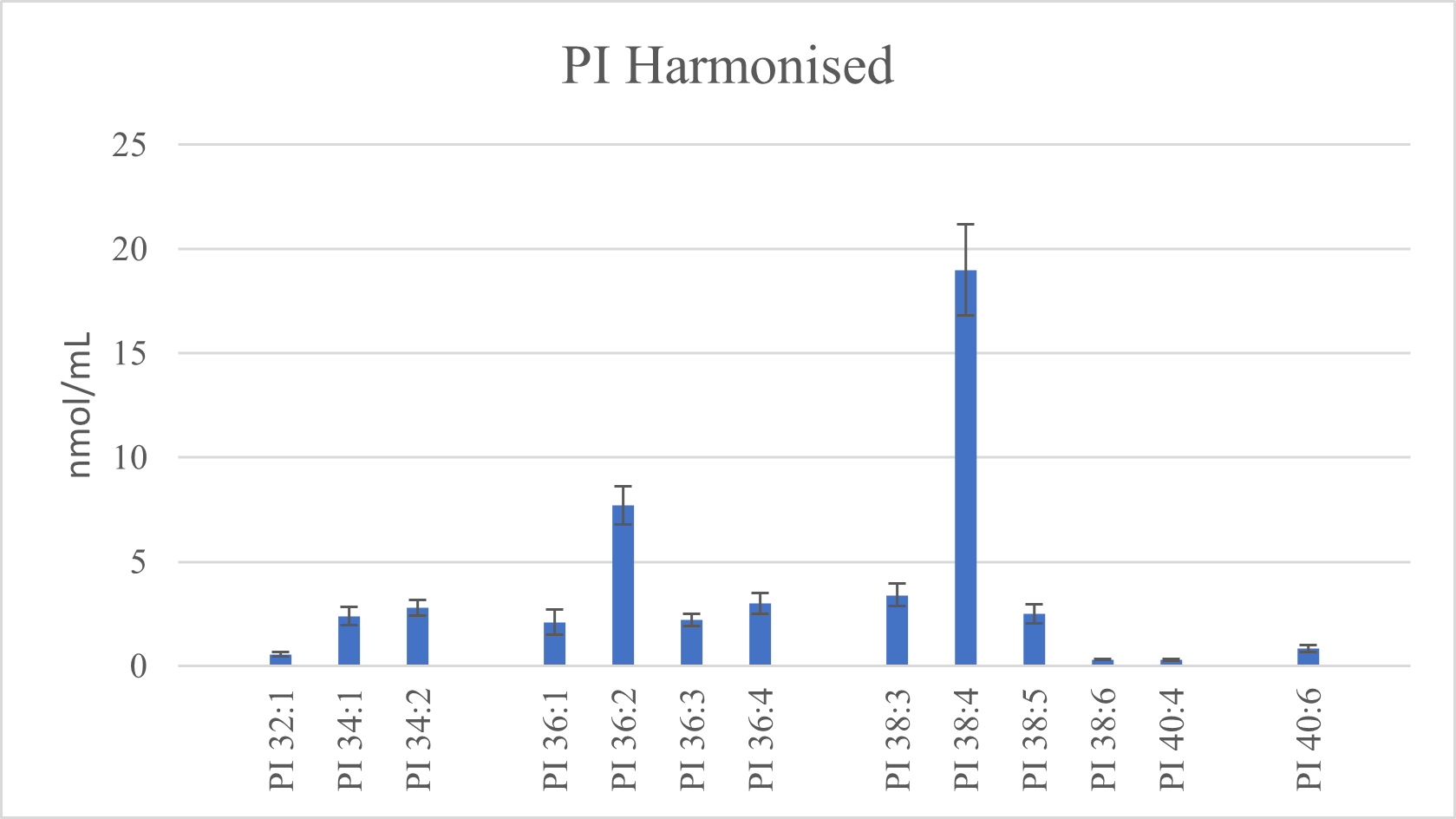


#


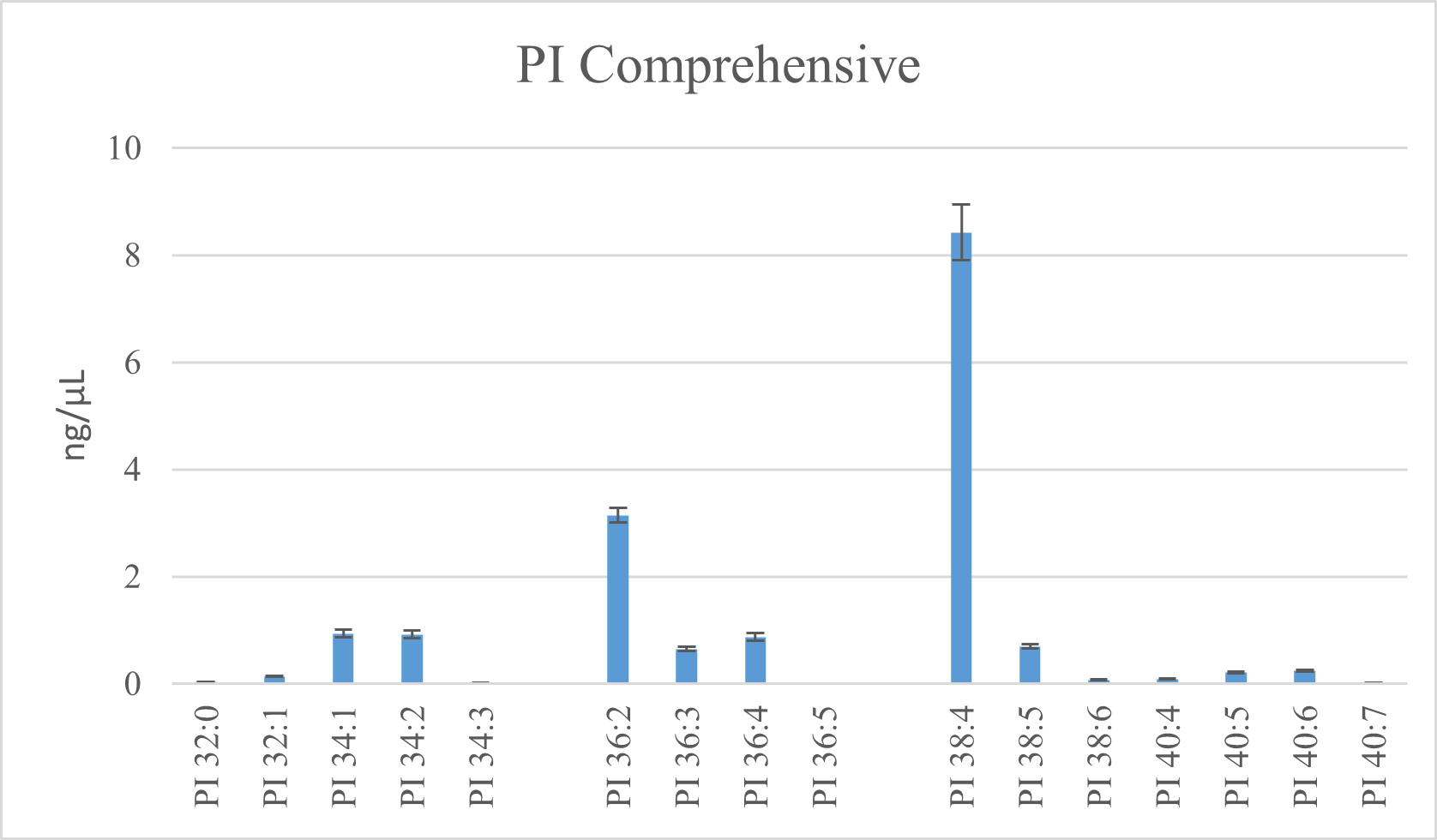


# Figure S-9. Phosphatidylinositol (PI) profile obtained in human plasma SRM 1950: PI comprehensive are the results produced with the GP method described here. PI harmonised are the results obtained by Bowden *et al.*

*
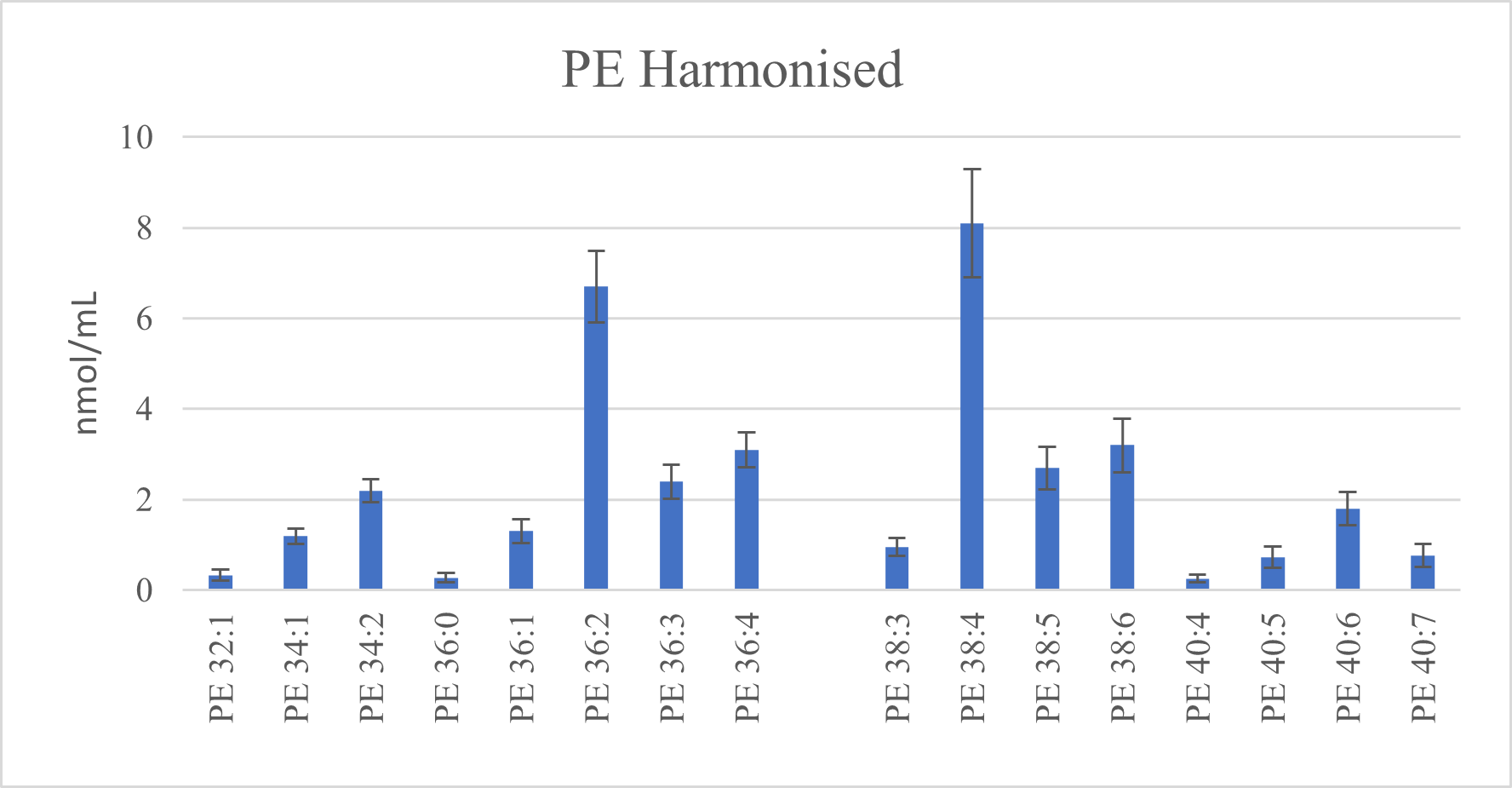
*

*
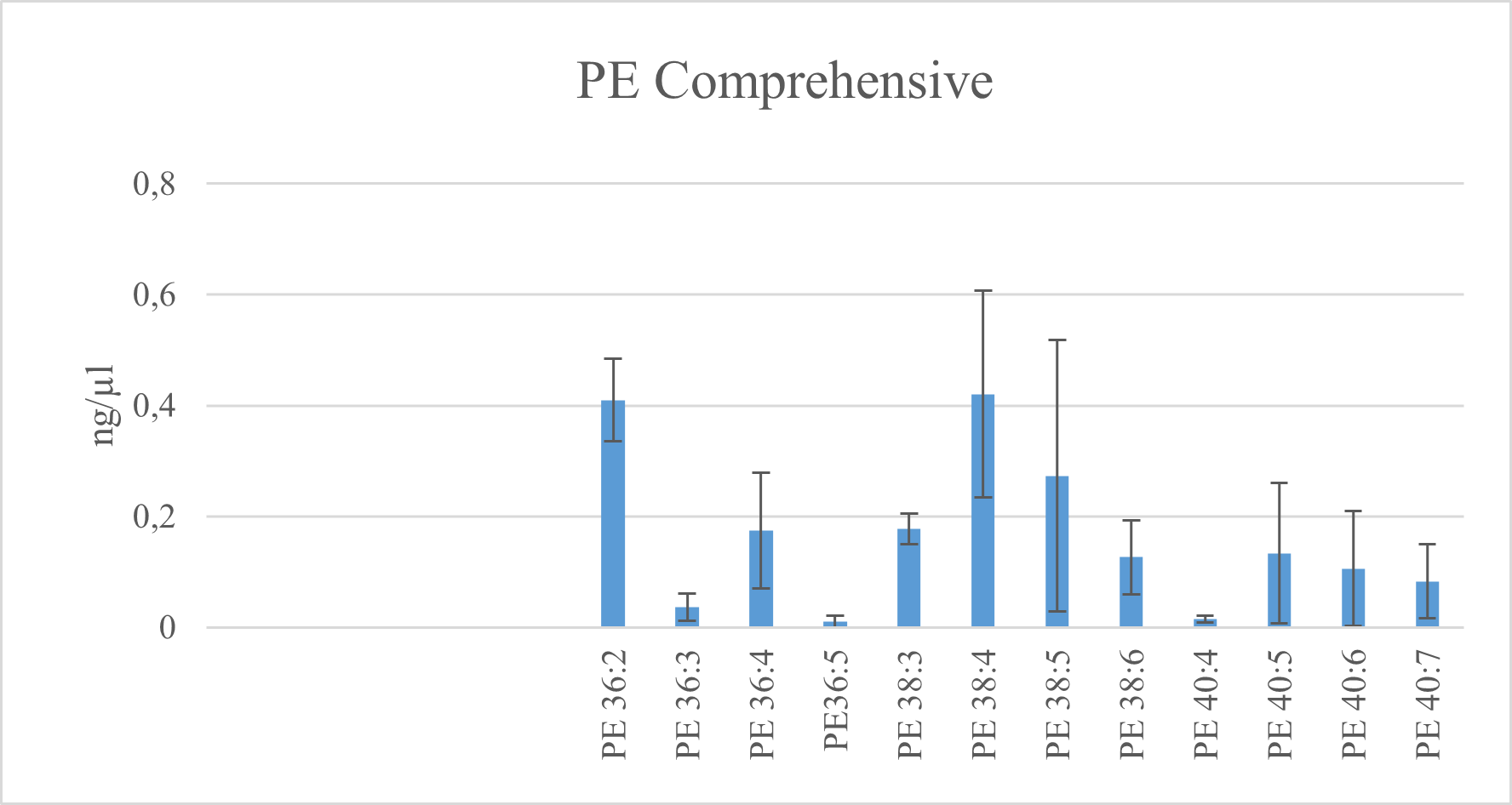
*

# Figure S-10. Phosphatidylethanolamine (PE) profile obtained in human plasma SRM 1950: PE comprehensive are the results produced with the GP method described here. PE harmonised are the results obtained by Bowden *et al.*


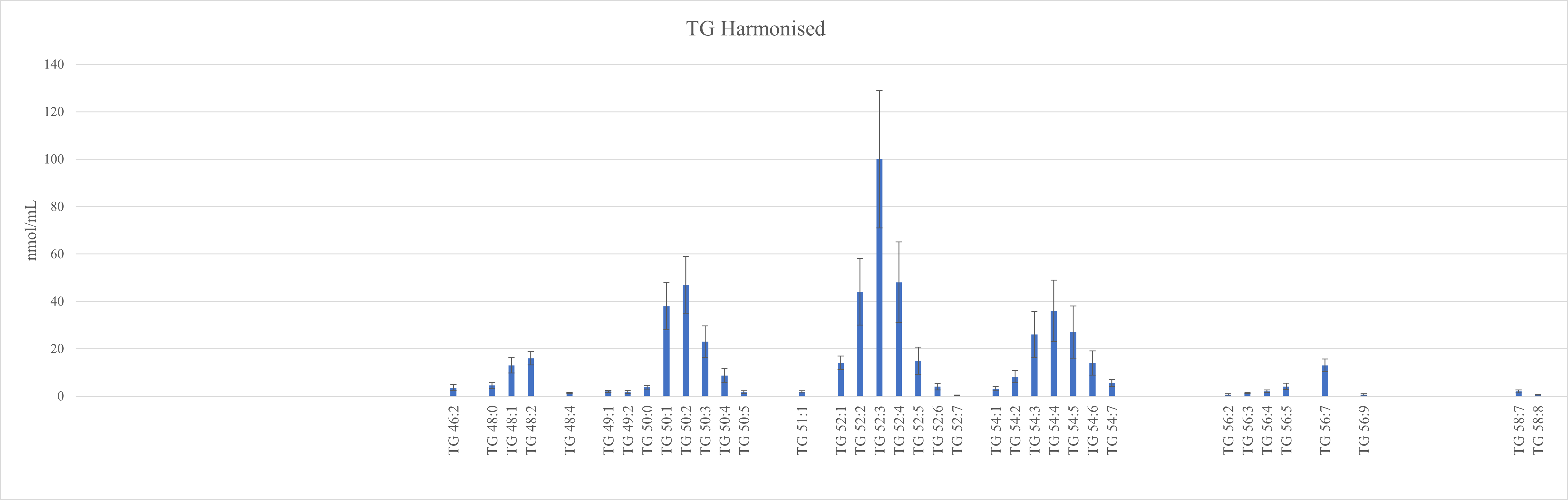


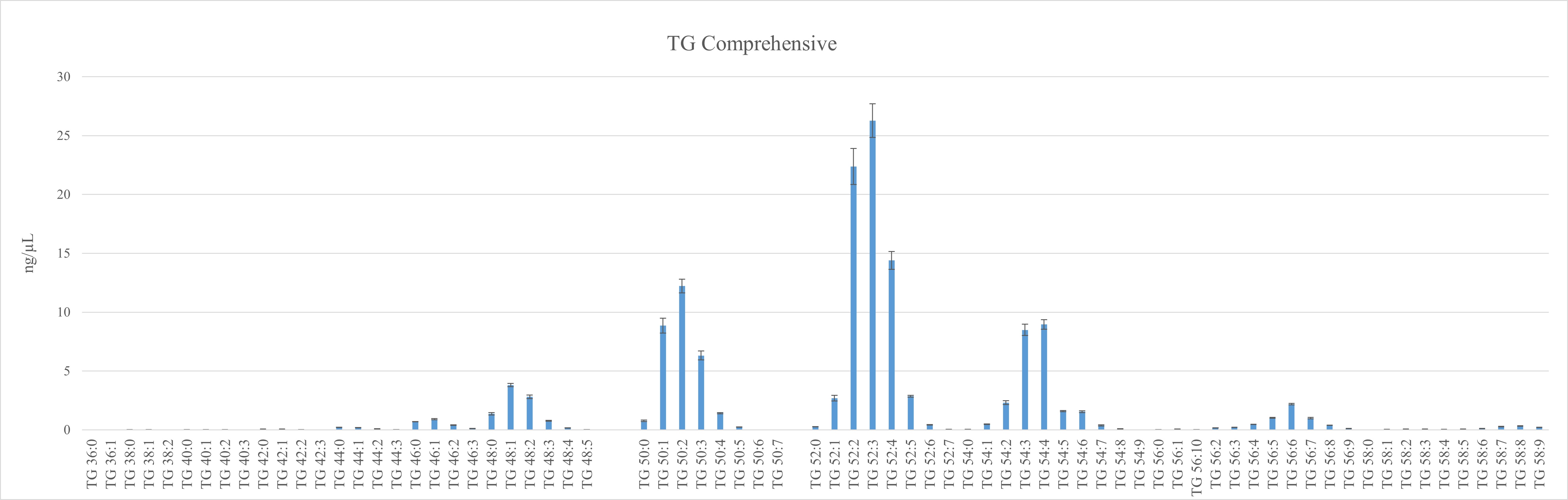


# Figure S-11. Triacylglycerols (TG) profile obtained in human plasma SRM 1950: TG comprehensive are the results produced with the GP method described here. TG harmonised are the results obtained by Bowden *et al.*

*
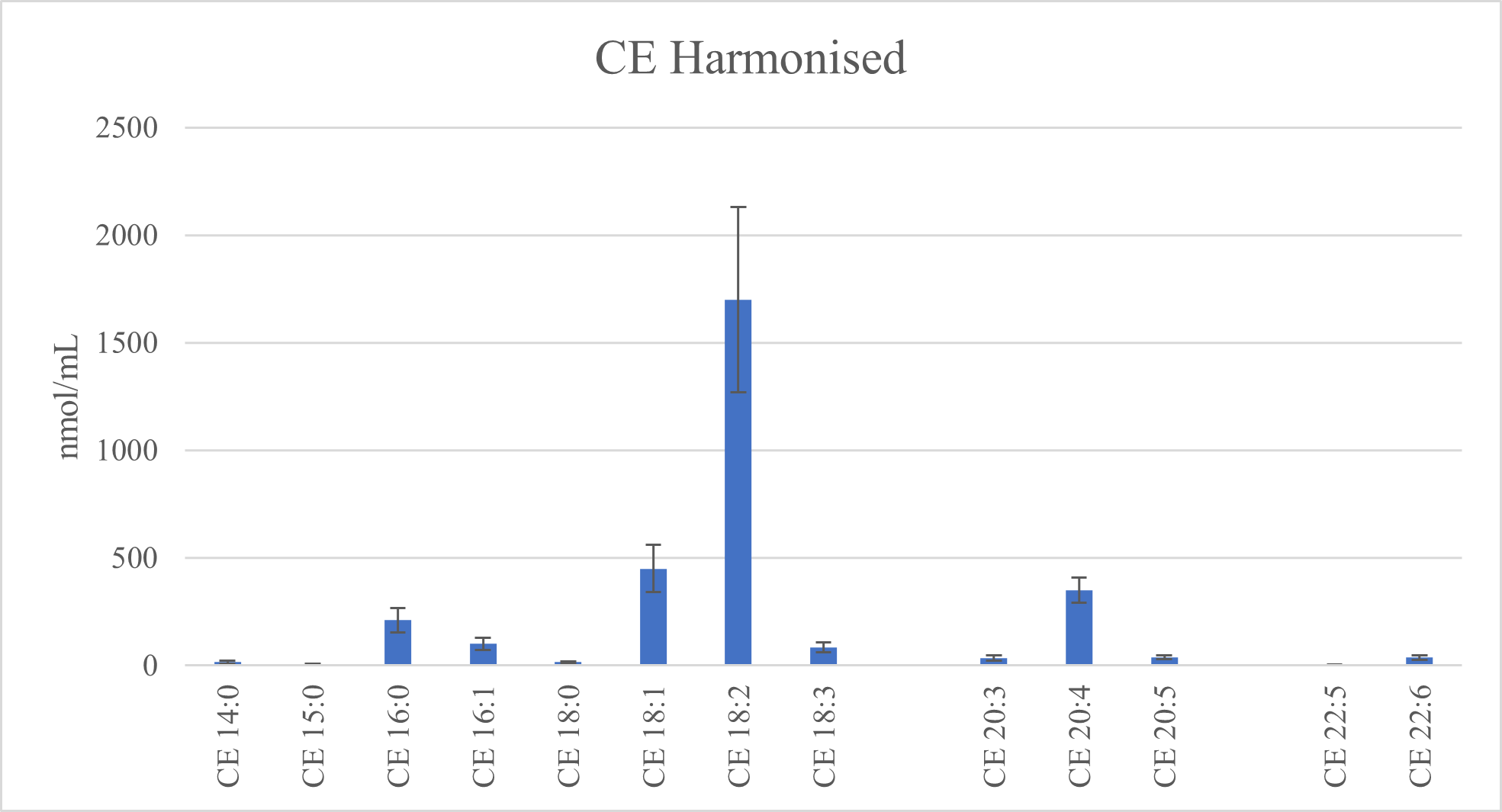
*


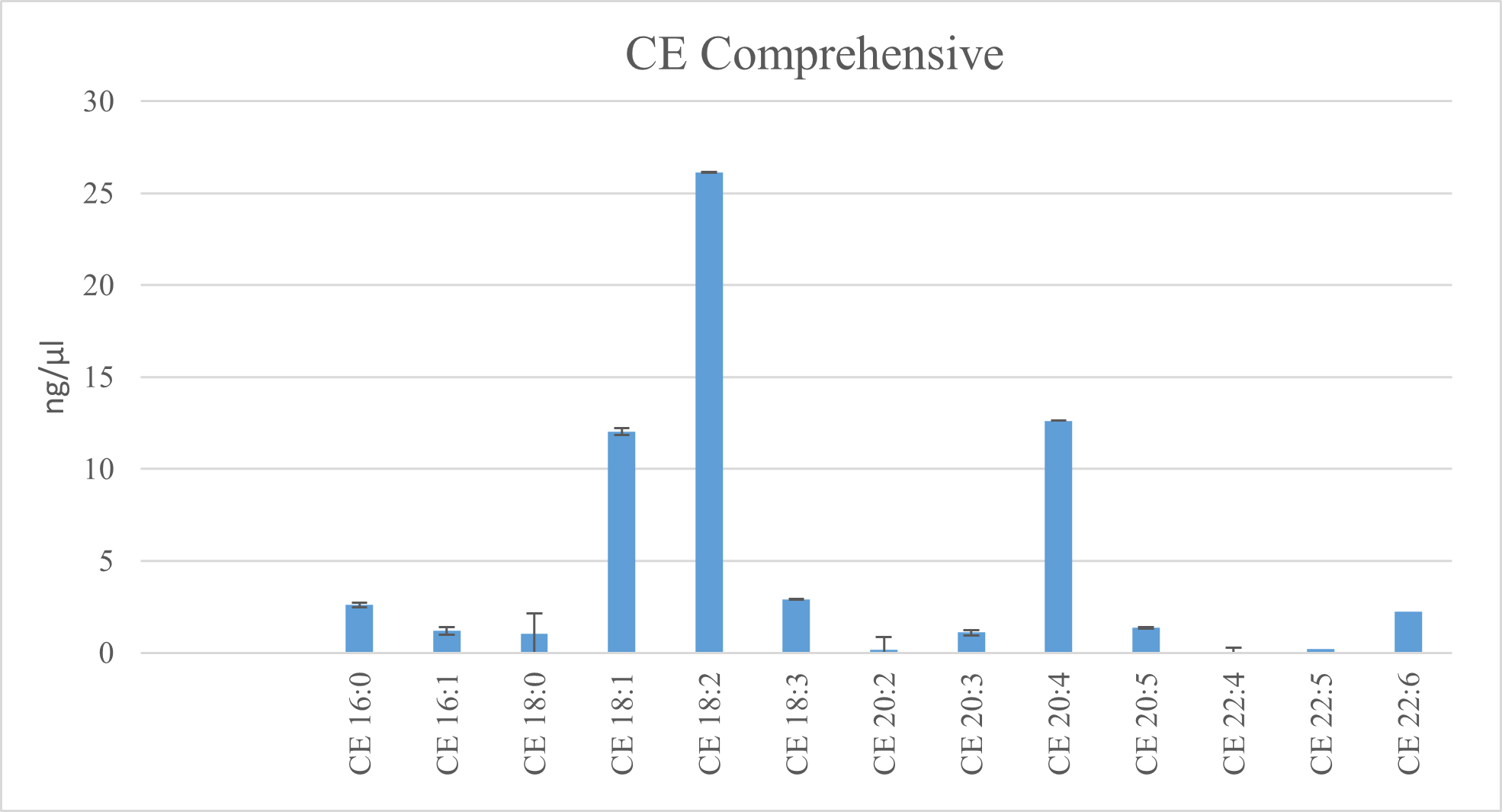


# Figure S-12. Cholesteryl ester (CE) profile obtained in human plasma SRM 1950: CE comprehensive are the results produced with the GP method described here. CE harmonised are the results obtained by Bowden *et al.*


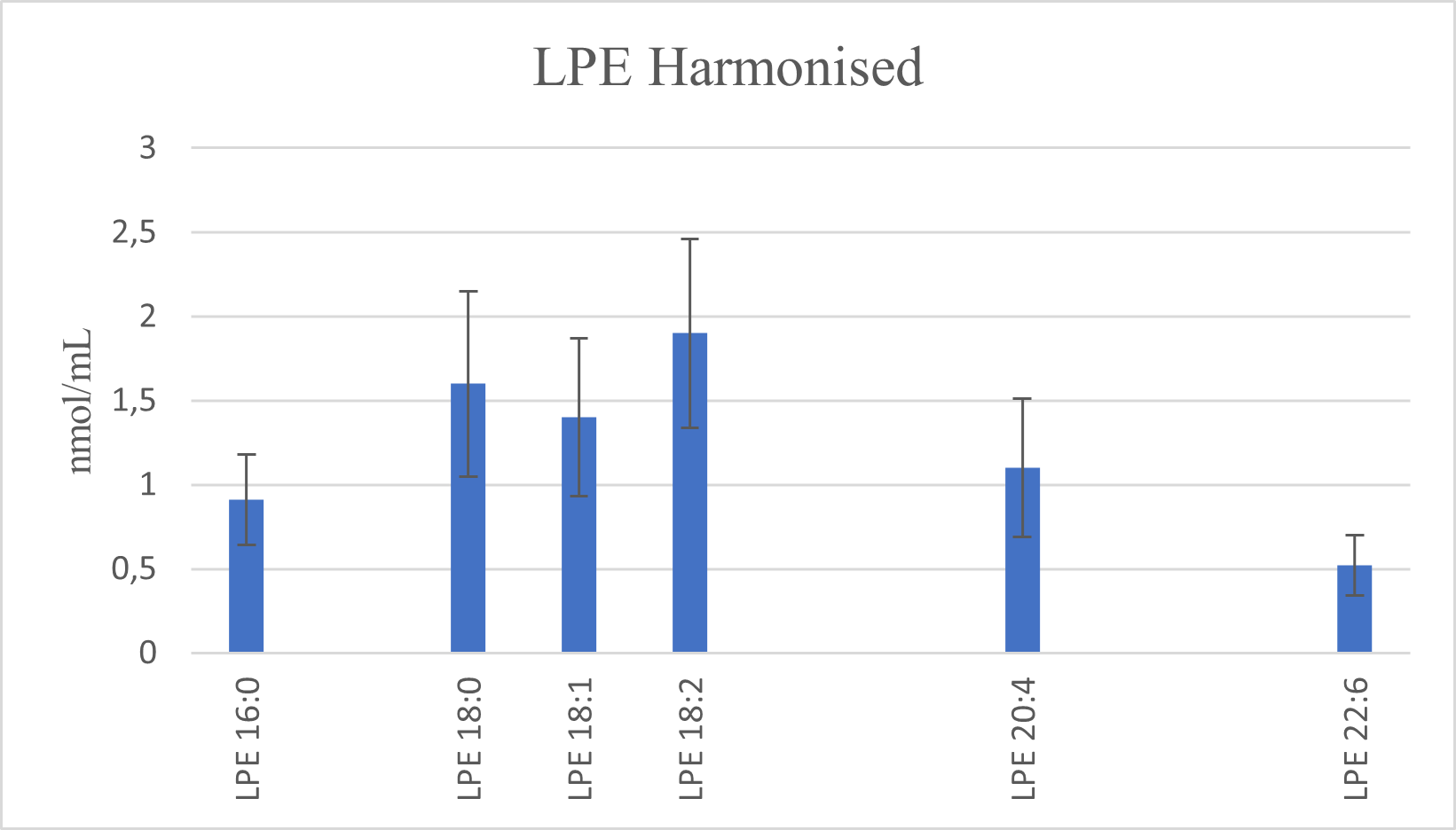


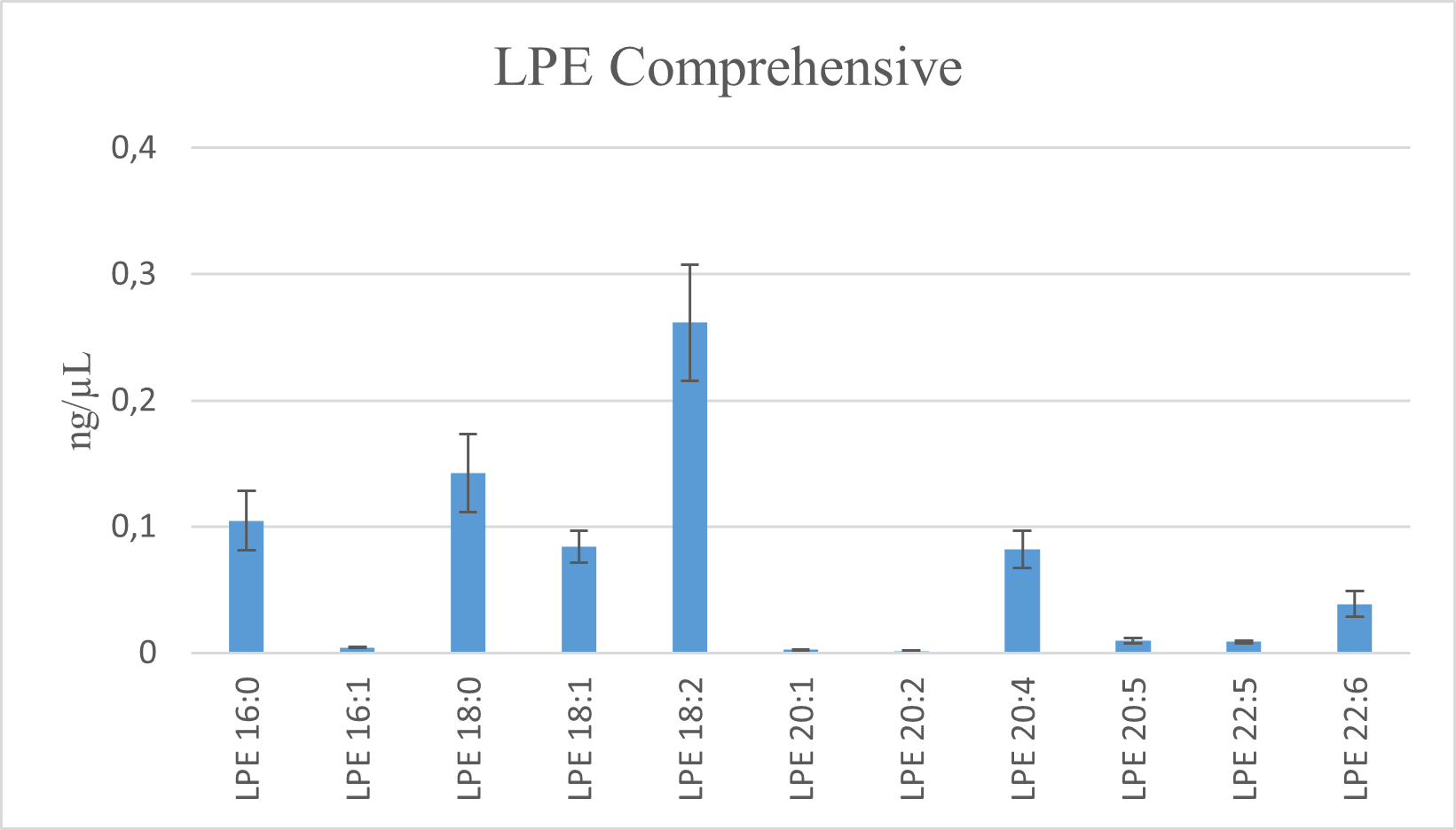


# Figure S-13. LPE profile obtained in human plasma SRM 1950: LPE comprehensive are the result produced with the GP method described here. LPE harmonised are the results obtained by Bowden *et al.*
